# Problems when fixing the response bias parameter *z* in drift diffusion analysis: A Commentary on Stafford et al. (2020)

**DOI:** 10.3758/s13428-021-01786-0

**Published:** 2022-03-22

**Authors:** Rainer W. Alexandrowicz, Bartosz Gula

**Affiliations:** Institute for Psychology, University of Klagenfurt, Universitaetsstrasse 65, 9020 Klagenfurt, Austria

**Keywords:** Drift diffusion modeling, Speed–accuracy trade-off, EZ, Estimation bias

## Abstract

In a simulation study, Stafford et al. (*Behavior Research Methods, 52*, 2142–2155, 2020) explored the effect of sample size on detecting group differences in ability in the presence of speed–accuracy trade-offs using the Drift Diffusion Model (DDM) and introduced an online tool to perform a power analysis. They found that the DDM approach was superior to analyzing the observed response times and response accuracies alone. In their simulation, they applied the EZ method to estimate the model parameters. In this article, we demonstrate that the EZ method, which cannot estimate the response bias parameter of the DDM, causes severe estimation bias for all parameters if the true response bias is not 0.5. Moreover, the bias patterns differ between EZ and the equivalent maximum likelihood estimation with *z* fixed at 0.5. This should be taken into consideration when using the otherwise excellent power analysis tool for experimental designs, in which *z*≠ 0.5 cannot be ruled out or even stipulate it.

**Supplementary Information:**

The online version contains supplementary material available at 10.3758/s13428-021-01786-0.

## Introduction

When asked for a decision, we have to choose whether to respond rather fast or rather accurate. This speed–accuracy trade-off (SATO; see Heitz, 2014; Henmon, 1911) is a well-established phenomenon in decision-making experiments. Generally, both response time (RT) and response accuracy (i. e., proportion correct) decrease when participants emphasize speed over accuracy. However, evaluating just one of these two outcome variables will not fully exhaust the available information. One way to overcome this limitation is to create an index combining both RT and accuracy (e. g., Bruyer & Brysbaert 2011; Liesefeld & Janczyk 2019; Townsend & Ashby 1978; Vandierendonck 2017, 2018, 2021; Woltz & Was 2006). Another way is to apply a model accounting for both measures. One such model is the Drift Diffusion Model (DDM; Ratcliff, 1978). It was successfully applied to RTs allowing for simultaneously considering RT and response accuracy and thus obtain a deeper understanding of the processes underlying the outcome (Voss, Rothermund, & Voss, 2004).

Basically, the DDM assumes in a two alternative forced choice (2AFC) situation the respondent to accumulate evidence in favor of either response option. Two thresholds indicate boundaries eventually hit after sufficient evidence has been collected for the respective decision, inducing a manifest reaction. This process is modeled with four main parameters: *(i)* The threshold distance (or separation) *a* indicates the amount of evidence required to issue a reaction. *(ii)* The drift rate (or drift parameter) *ν* is the average rate of evidence accumulation per time unit. *(iii)* The response bias parameter *z* covers the respondent’s initial expectation, which decision is likely to be taken next (e. g., by instructing participants that 80 % of the stimuli will require a positive response). *(iv)* Additionally, all time components not related to forming the decision (i. e., encoding the stimulus and executing the response) are aggregated in the encoding and response time parameter *t*_ER_. These four parameters were later supplemented by three more parameters covering also inter-trial variability of *z*, *ν*, and *t*_ER_ (Ratcliff & Rouder, 1998). Additionally, the intra-trial variability parameter of *ν* (frequently termed *s*^2^) is set (usually to a value of 1 or 0.1) to make the model identified. From these parameters, the drift parameter *ν* has proven to reflect the efficiency of stimulus processing and the threshold separation *a* the speed–accuracy response settings. Alexandrowicz (2020) presented the DMV (Diffusion Model Visualizer), an interactive graphical tool, which helps understanding how these model parameters form the RT distributions at either boundary and the response probabilities at either boundary. By means of sliders for all eight model parameters^1^, the user can interactively change each parameter and instantaneously see how this impacts the resulting RT densities and response probabilities. The tool is especially helpful for understanding SATO and the role the two core parameters *a* and *ν* play therein.

The model parameters are estimated by evaluating the response-time distributions and proportions of hits at either threshold. A variety of estimation methods has been developed and compared (e. g., Alexandrowicz and Gula (2020), Arnold et al., (2015), Dutilh et al., (2019), Lerche and Voss (2018), and Ratcliff and Tuerlinckx (2002)).

In a simulation study, Stafford, Pirrone, Croucher, and Krystalli (2020) explored the superiority of a DDM approach over analyzing response time and accuracy separately. They simulated RT and accuracy data with a DDM by assuming two groups in a no-difference condition and a SATO-condition, the latter realized by varying both the boundary separation parameter *a* and the drift parameter *ν*. They analyzed the differences of speed and accuracy conditions using *(a)* only RTs, *(b)* only accuracy, and *(c)* the combined evaluation with a DDM. Moreover, they compared the effect the number of participants (*n*) had on the results of these three approaches, assuming 40 trials per person. The results of the DDM (i. e., the drift parameter), which takes into account both RTs and response accuracy, outperformed the separate evaluation of the two measures. This was particularly the case in the presence of SATOs. The authors found that only the drift rate was robust in the presence of SATOs, whereas response speed and accuracy alone produced a large number of false positives. They emphasized the gain in power in detecting group differences with the DDM and introduced the “decision poser” (Krystalli & Stafford, 2019), an online tool allowing for performing an a priori power analysis for determining the optimal sample size required to detect relevant differences in drift between two groups.

## Problem

Stafford et al., (2020) used in their simulation the EZ method (Wagenmakers, van der Maas, & Grasman, 2007) to estimate the model parameters for its advantage in computational speed. In contrast to all other estimation methods, EZ is a closed form algorithm, employing the mean, the variance and the proportion of responses at the upper threshold, which makes it extremely fast in contrast to ML-based methods. However, it has one drawback as it does not allow for estimating the response bias *z* but rather fixes this parameter at *a*/2 (i. e., assumes that there is no response bias).

In a comprehensive simulation study, Alexandrowicz and Gula (2020) compared eight methods for estimating the parameters of the DDM with respect to parameter recovery. These were the EZ algorithm, several maximum likelihood methods, and an implementation following the Bayesian principle. One core result was that all methods performed fairly equally in recovering the original parameters used for simulating the data sets. However, aside of EZ’s inability to estimate the response bias, it also caused severe estimation bias and larger RMSE of *a*, *ν*, and *t*_ER_ in several settings.

Stafford et al., (2020) reported that they checked their results they obtained with EZ against the hierarchical HDDM method (Wiecki, Sofer, & Frank, 2013) and fast-dm (Voss and Voss, 2007). However, they do not go into details regarding this comparison, especially, which parameter constraints were applied in the HDDM and fast-dm estimations: These methods support a broader approach, allowing for estimating not only the response bias parameter *z*, but also three additional parameters covering the inter-trial-variability of *ν*, *z*, and *t*_ER_. If they set these variability parameters to zero (as is implicitly done in the EZ-routine), the similarity is expectable. But if these additional parameters were also estimated freely with HDDM, it would seem unlikely to obtain similar results. Unfortunately, the authors only state “We also confirm that the basic pattern of results holds for […] the HDDM […] and fast-dm” (Stafford et al., 2020, p. 2145), which is too vague for a clear statement on this issue.

The biases associated with the EZ method found by Alexandrowicz and Gula (2020) along with the somewhat unclear presentation of Stafford et al., (2020) raise the question, whether EZ is an appropriate method to perform the power analysis as proposed by Krystalli and Stafford (2019). This aspect is explored below.

## The consequences of fixing the response bias parameter *z*

For that purpose, we performed a simulation study, in which we generated data sets in line with the DDM applying a full grid search across a wide range of model parameter values. We estimated the four model parameters (*a*, *z*, *ν*, and *t*_ER_) for each simulated data set with (a) the unrestricted ML-method, (b) the ML-method with *z*_rel_ fixed at 0.5, and (c) the EZ-method. The Supplement provides details regarding the simula-tion technique, the chosen simulation parameters, and the results. The core results will be presented and discussed here.

### Comparing parameter recovery across methods

**Fig. 1:**
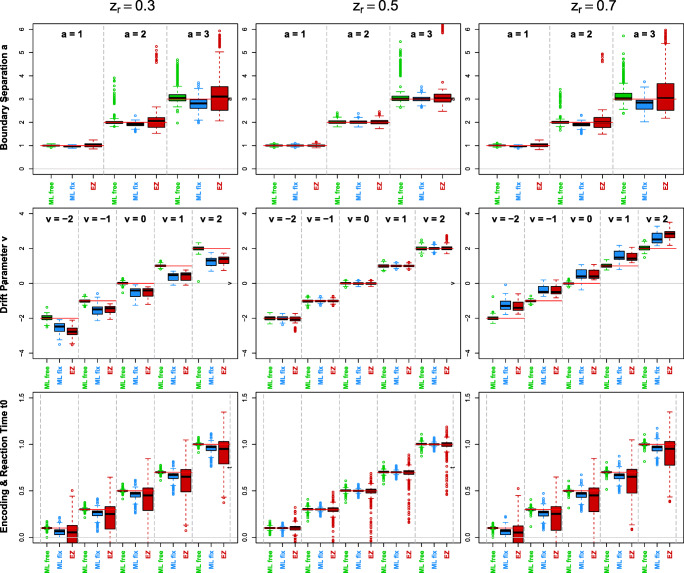
Boxplots of parameter estimates $\hat {a}$, $\hat {\nu }$, and $\hat {t}_{0}$ split by *z* and estimation method (ML: *green*, restricted ML: *blue*, EZ: *red*). The *horizontal red lines* indicate the original values of each parameter

Figure 1 shows the parameter recovery of *a*, *ν*, and *t*_ER_ broken down for the chosen levels of *a*, *ν*, and *z*_rel_.

Considering the core parameter *z*, the middle column of Fig. 1 shows the estimates of *a*, *ν*, and *t*_ER_ for *z*_rel_ = 0.5. All three methods provide unbiased parameter estimates, but EZ exhibits large positive outliers for *a* and large negative outliers for *t*_ER_ (the latter even falling below zero for true *t*_ER_ ≤ 0.5).

In contrast, we face estimation problems when switching to *z*_rel_≠ 0.5. In the first column (*z*_rel_ = 0.3), fixed ML and EZ underestimate both *ν* and *t*_ER_. A detailed analysis revealed that this was the case for large |*ν*|, which caused the process to hit only the upper or the lower boundary (depending on the sign of *ν*). In the third column (*z*_rel_ = 0.7), the fixed ML method shows a tendency to underestimate *a* and to overestimate *ν*. The EZ method overestimates *ν* and underestimates *t*_ER_, with the latter even yielding invalid estimates of *t*_ER_ < 0.

### The free ML Parameter estimation method

**Fig. 2:**
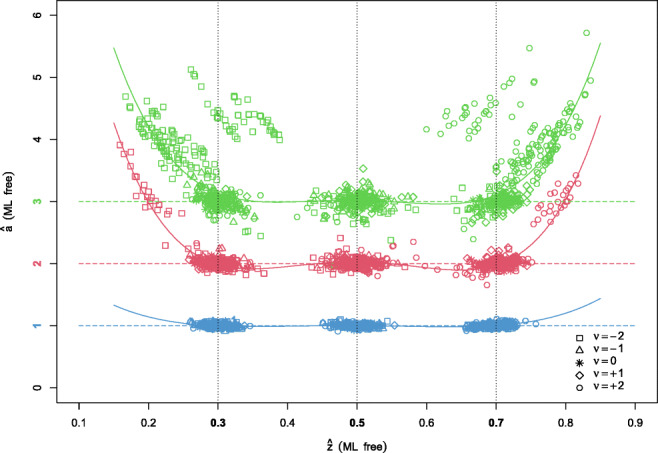
Estimates $\hat {a}$ (vertical axis) for random fluctuations of $\hat {z}$ (*horizontal axis*). The *colors* and the *horizontal dashed lines* indicate the true values of *a* (*blue*: *a* = 1, *red*: *a* = 2, *green*: *a* = 3), the *vertical dotted lines* indicate the true values of *z* and the shapes indicate the true values of *ν* (see legend); the *solid lines* indicate a fourth-degree polynomial approximation

Generally, the free ML method (colored green) did not show any bias across any level of *z*_rel_ in Fig. 1 except for some outliers for large values of *a*, which is analyzed below. Figure 2 reveals an interesting interaction effect of $\hat {a}$, $\hat {\nu }$, and $\hat {z}$.

**Fig. 3:**
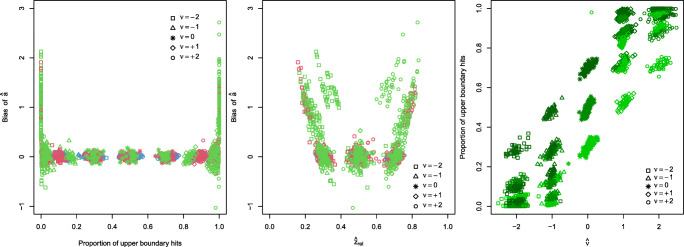
*Left diagram*: relating the bias of $\hat {a}$ to the proportion of upper boundary hits (colors indicate the levels of *a*, see Fig. 2); *middle diagram*: relating the bias of $\hat {a}$ to the estimates $\hat {z}_{\text {rel}}$ (*colors* indicate the levels of *a*, see Fig. 2); *right diagram*: relating the proportion of upper boundary hits to the drift parameter estimates $\hat {\nu }$ (*colors* indicate the levels of *z*: *light green*: *z* = 0.3, *medium green*: *z* = 0.5, *dark green*: *z* = 0.7)

The horizontal spread of the elliptical clusters shows the random fluctuations of the sample estimates $\hat {z}$ (see also Fig. 9 in the Supplement). These together with larger absolute values of *ν* (indicated by squares for *ν* = + 2 and circles for *ν* = − 2) cause the upwards biased estimates $\hat {a}$. The effect even increases with *a*. As a rough approximation, a fourth-degree polynomial was fitted for each *a* indicating a noticeable agreement to the upwardly biased estimates $\hat {a}$ for extreme $\hat {z}$. Figure 3 illustrates the mechanism behind this bias

The bias of $\hat {a}$ appears only for data sets, in which one of the two boundaries is (almost) never hit (left diagram of Fig. 3), which occurs for extreme values of $\hat {z}_{\text {rel}}$ (middle diagram), which, in turn, is related to extreme values of |*ν*| (right diagram). For applications, we should, therefore, keep in mind that data sets with no or almost no hits at one of the two boundaries may be slightly affected by the bias described here. However, because such data sets arise under rather uncommon parameter constellations and would be easily detected in empirical data, we conclude that the ML free method is entirely unproblematic.

### Fixing *z* at 0.5

Figure 1 shows some severe biases for both methods fixing *z* at 0.5 (i. e., ML fix and EZ). However, this figure does not yet reveal the entire complexity of the structure of these biases. Rather, we have to inspect the estimates per subgroup formed by the various parameter combinations. The Supplement provides a full breakdown, the core results of which are summarized here followed by certain additional analyses. We found that

- both ML fix and EZ
  - severely underestimate *ν* for *z* < 0.5 and severely overestimate it for *z* > 0.5 (see Fig. 1 here / Fig. 12 in the Supplement);
  - severely underestimate *t*_ER_ for *z*≠ 0.5, EZ even yielding estimates below zero (see Fig. 1 here / Fig. 12 in the Supplement);
- further, ML fix
  - severely underestimates *a*, the more the larger *a* and increasingly for *ν* < 0/*z* < 0.5 and *ν* > 0/*z* > 0.5 (see Figure 14 in the Supplement);
  - severely underestimates $\hat {\nu }$ for *z* < 0.5 and overestimates it for *z* > 0.5 (see Figure 17 in the Supplement);
  - underestimates *t*_ER_ for *z*≠ 0.5 the more the larger *a* and the smaller |*ν*| (see Figure 20 in the Supplement);
- and EZ
  - severely misestimates *a*, increasingly for larger *a*. The direction depends on the combination of *z* and *ν*, as the following table illustrates:

**Table:** 

EZ	*ν* < 0	*ν* > 0
*z* < 0	*a* overestimated	*a* underestimated
*z* > 0	*a* underestimated	*a* overestimated
    (see Fig. 15 in the Supplement);
  - severely underestimates $\hat {\nu }$ for *z* < 0.5 and overestimates it for *z* > 0.5 but the more the *smaller* *a* (see Fig. 18 in the Supplement);
  - misestimates *t*_ER_ for *z*≠ 0.5 entirely and the more the larger *a*. The bias has the following pattern:

**Table:** 

EZ	*ν* < 0	*ν* > 0
*z* < 0	*t*_ER_ underestimated	*t*_ER_ overestimated
*z* > 0	*t*_ER_ overestimated	*t*_ER_ underestimated
    (see Figure 21 in the Supplement);

To exemplify the specific problems of EZ, Fig. 4 juxtaposes the estimates $\hat {a}$ for ML fix and EZ.

**Fig. 4:**
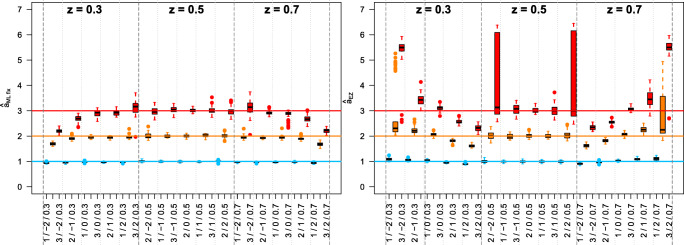
Estimates $\hat {a}_{\mathrm {ML~fix}}$ (left diagram) and $\hat {a}_{\text {EZ}}$ by *z* × *a* × *ν*; (Figs. 14 and 15 from the Supplement, see there for details)

Clearly, the two estimation methods show an opposite structure of bias, with ML fix following a reversed U-shape and EZ a U-shape. Diverging structures for ML fix vs. EZ also emerged for $\hat {\nu }$ (see Figs. 17 and 18 in the Supplement) and $\hat {t}_{\text {ER}}$ (Figs. 20 and 21). This indicates that the problem is not only the fixing of *z* = 0.5 as such, but that more subtleties are in effect, with EZ performing inferior (or even erratic) in all instances.

### Practical implications

The simulation results relate to the two-group setting of Stafford et al., (2020) as follows: Suppose the two groups differed in response bias and drift rates were positive. Estimates from EZ and ML fixed would both lead not only to different but also wrong conclusions about SATOs: EZ estimates would imply that participants in the group with the larger bias were more cautious than those in the group with the relatively smaller bias (see Fig. 4/right panel and Fig. 14 in the Supplement). In contrast, the ML fix estimates would imply that the latter participants were more cautious (see Fig. 4/left panel and Fig. 14 in the Supplement). Additionally, both estimation methods would incorrectly “translate” a true between-group difference in bias into an effect in drift rate (see Section 5.2 in the Supplement). Hence, the potential of the DDM to isolate SATOs from true between-group effects comes at the price of an increased likelihood for false-positive conclusions about the presence of SATOs if response bias is neglected.

## Discussion

With the present simulation study, we demonstrated that tampering with *z* when estimating the parameters of a DDM has a strong and detrimental impact on the other parameters’ estimates. Stafford and colleagues fixed *z* at *a*/2 in their simulations in order to prevent estimation bias due to model misspecification. However, we could show that any procedure fixing *z* implicitly (EZ) or explicitly (ML fix) will most likely result in biased estimates of *a*, *ν*, and *t*_ER_.

This is of practical relevance because we have to expect *z* to differ in many experiments: On the one hand, response bias has been shown to be sensitive to manipulations affecting the expectation which response is better, such as proportion of stimuli, pay-offs, or features of preceding stimuli (Diederich and Busemeyer, 2006; Simen et al., 2009; Burnham, 2018). On the other hand, *z* may also be particularly important from a theoretical point of view, such as in memory (Starns et al., 2012; White & Poldrack, 2014) or stereotype research (Johnson, Cesario, & Pleskac, 2018; Mayerl, Alexandrowicz, & Gula, 2019). And, as we could show in our simulation, purely random fluctuations of *z*, that we can never rule out, may also result in an estimation bias, especially for large *a* and *ν*. In an experiment targeting the SATO, this will be the case in an accuracy condition (i. e., large *a*) when presenting “easy” stimuli (i. e., large |*ν*|). In contrast, it will not happen in a speed condition or when using “difficult” stimuli. Hence, the EZ method used by Stafford and colleagues may not always control well for speed–accuracy trade-offs.

In their study, Stafford et al., (2020) demonstrate convincingly the advantages of a model-based approach to analyze response time and response accuracy in a two-alternatives forced-choice experiment over analyses of either measure alone. Moreover, they explore the important question of how many participants are required to detect group differences in speed and accuracy with a given probability of errors of the first and second kind and introduce a handy online tool to perform such a power analysis. Their choice of EZ to estimate the DDM parameters was a comprehensible decision, for any other method would have been prohibitive for their endeavor. However, this study again demonstrated the weaknesses of the EZ method. First and foremost, it is only applicable if no response bias is present. Wagenmakers et al., (2007) already noted that “When such a bias exists, the “vanilla” version of the EZ-diffusion model presented here is inappropriate.” (p. 8). Also, Grasman, Wagenmakers, and van der Maas (2009) pointed out that certain experimental designs cannot be covered by the assumptions made when applying the EZ method (e. g., in a lexical decision paradigm with conditions and correctness intertwined; p. 55). Ratcliff (2008) used the term “misspecification” (p. 1224) and Wagenmakers, van der Maas, Dolan, and Grasman (2008) conceded that the *z* = *a*/2 assumption “may be overly restrictive” (p. 1230). Further, Liesefeld and Janczyk (2019) showed that limiting the response time (either by experimental design through a response deadline or by trimming RTs considered as outliers) may result in over-estimating *t*_ER_ in cases, in which *a* is large (p. 55). We therefore argue that the systematic over- or underestimation of the boundary separation parameter *a* and drift rate *ν* renders EZ not an adequate method for SATO research, even less for designs stipulating *z*≠*a*/2.

One approach to check the plausibility of the assumption that *z* = 0.5 would be to examine the mean RTs at each boundary, and, if they differ, to estimate *z* instead (see Wagenmakers et al.,, 2007, for further checks for misspecification). Moreover, we showed that even if there is no response bias, fixing *z* will in certain cases result in biased estimates of *ν* and (to an even larger extent) of *t*_ER_. Therefore, even in studies not explicitly triggering response bias it seems advisable to estimate *z*. Nevertheless, and despite the shortcomings discussed here, the “decision power” tool (Krystalli & Stafford, 2019; Stafford et al., 2020) fills a gap in current research methods.

Interestingly, van Ravenzwaaij and Oberauer (2009) found in their simulation study EZ outperforming the fast-dm (i. e., ML-based) estimation method, which seems to contradict the present results. However, their simulation design was benevolent with respect to EZ in that they assumed *z* = *a*/2 (p. 465) and only considered a selected set of true parameters mirroring estimates from one specific experiment (even further adjusted by hand; p. 466). Similarly, van Ravenzwaaij et al., (2017) found EZ to perform well and comparable to the DDM. However, they focused (explicitly) on the power of detecting group differences rather than obtaining exact parameter estimates and either fixed *z* = 0.5 in three of their simulations or sampled it from an N(0.5,0.04), which yields *z* outside the interval (0.4,0.6) with a probability of less than 1.3 %. Hence, their conclusions also refer to designs with (almost) no bias (what they also point out in Footnote 3 on p. 551). In contrast, our grid-search approach is much wider and allows for identifying the structural weaknesses of EZ reported here. This applies especially – but not limited to – the negative $\hat {t}_{\text {ER}}$, which, to our knowledge, have not been addressed before, thus casting severe doubts on the adequacy of this model at all.

We therefore consider EZ as generally problematic, which is also in line with Alexandrowicz and Gula (2020), who recommend EZ preferably “for quickly obtaining suitable starting values”, not considering it “an equivalent alternative” for estimating the parameters of the DDM (p. 17). Maybe, we should take Wagenmakers et al., (2007) literally and consider EZ a *model* of its own rather than just another estimation method for the DDM parameters.

## Electronic supplementary material

Below is the link to the electronic supplementary material.

(PDF 1.29 MB)

## Footnotes

1 Note that in combination with this article, a new version of the DMV is made available at www.dmvis.at, now also supporting a slider for *s*^2^. The author RWA wishes to thank Heinrich René Liesefeld for encouraging to implement this extension.

## References

[CR1] Alexandrowicz RW (2020). The diffusion model visualizer: An interactive tool to understand the diffusion model parameters. Psychological Research.

[CR2] Alexandrowicz RW, Gula B (2020). Comparing eight parameter estimation methods for the Ratcliff Diffusion model using free software. Frontiers in Psychology.

[CR3] Arnold NR, Bröder A, Bayen UJ (2015). Empirical validation of the diffusion model for recognition memory and a comparison of parameterestimation methods. Psychological Research Psychologische Forschung.

[CR4] Bruyer R, Brysbaert M (2011). Combining speed and accuracy in cognitive psychology: Is the inverse efficiency score (IES) a better dependent variable than the mean reaction time (RT) and the percentage of errors (PE)?. Psychologica Belgica.

[CR5] Burnham BR (2018). Selection and response bias as determinants of priming of pop-out search: Revelations from diffusion modeling. Psychonomic Bulletin & Review.

[CR6] Diederich A, Busemeyer J (2006). Modeling the effects of payoff on response bias in a perceptual discrimination task: Bound-change, drift-rate change, or two-stage-processing hypothesis. Perception & Psychophysics.

[CR7] Dutilh G, Annis J, Brown SD, Cassey P, Evans NJ, Grasman RPPP, Donkin C (2019). The quality of response time data inference: A blinded, collaborative assessment of the validity of cognitive models. Psychonomic Bulletin & Review.

[CR8] Grasman RPPP, Wagenmakers E-J, van der Maas HLJ (2009). On the mean and variance of response times under the diffusion model with an application to parameter estimation. Journal of Mathematical Psychology.

[CR9] Heitz RP (2014). The speed-accuracy tradeoff: History, physiology, methodology, and behavior. Frontiers in Neuroscience.

[CR10] Henmon VAC (1911). The relation of the time of a judgment to its accuracy. Psychological Review.

[CR11] Johnson DJ, Cesario J, Pleskac TJ (2018). How prior information and police experience impact decisions to shoot. Journal of Personality and Social Psychology.

[CR12] Krystalli, A., & Stafford, T. (2019). Interactive web application accompanying paper ‘Quantifying the benefits of using decision models with response time and accuracy data’. Retrieved from https://figshare.shef.ac.uk/s/11f65856db28308644a4, 10.15131/shef.data.8109161. 10.3758/s13428-020-01372-w PMC7575468 32232739

[CR13] Lerche V, Voss A (2018). Speed-accuracy manipulations and diffusion modeling: Lack of discriminant validity of the manipulation or of the parameter estimates?. Behavior Research Methods.

[CR14] Liesefeld HR, Janczyk M (2019). Combining speed and accuracy to control for speed-accuracy trade-offs(?). Behavior Research Methods.

[CR15] Mayerl H, Alexandrowicz RW, Gula B (2019). Modeling effects of newspaper articles on stereotype accessibility in the shooter task. Social Cognition.

[CR16] Ratcliff R (1978). A theory of memory retrieval. Psychological Review.

[CR17] Ratcliff R (2008). The EZ diffusion method: Too EZ?. Psychonomic Bulletin & Review.

[CR18] Ratcliff R, Rouder JN (1998). Modelling response times for two-choice decisions. Psychological Science.

[CR19] Ratcliff R, Tuerlinckx F (2002). Estimating parameters of the diffusion model: Approaches to dealing with contaminant reaction times and parameter variability. Psychonomic Bulletin & Review.

[CR20] Simen P, Contreras D, Buckand C, Huand P, Holmes P, Cohen JD (2009). Reward rate optimization in two-alternative decision making: Empirical test of theoretical predictions. Journal of Experimental Psychology: Human Perception & Performance.

[CR21] Stafford T, Pirrone A, Croucher M, Krystalli A (2020). Quantifying the benefits of using decision models with response time and accuracy data. Behavior Research Methods.

[CR22] Starns JJ, Ratcliff R, White CN (2012). Diffusion model drift rates can be influenced by decision processes: An analysis of the strength-based mirror effect. Journal of Experimental Psychology: Learning, Memory, and Cognition.

[CR23] Townsend, J.T., & Ashby, F.G. (1978). Methods of modeling capacity in simple processing systems. In N.J. Castellan, & F. Restle (Eds.) *Cognitive Theory 3* (pp. 199–239). New York: Lawrence Erlbaum Associates.

[CR24] Vandierendonck A (2017). A comparison of methods to combine speed and accuracy measures of performance: A rejoinder on the binning procedure. Behavior Research Methods.

[CR25] Vandierendonck A (2018). Further tests of the utility of integrated speed-accuracy measures in task switching. Journal of Cognition.

[CR26] Vandierendonck A (2021). On the utility of integrated speed-accuracy measures when speed-accuracy trade-off is present. Journal of Cognition.

[CR27] van Ravenzwaaij D, Donkin C, Vandekerckhove J (2017). The EZ diffusion model provides a powerful test of simple empirical effects. Psychonomic Bulletin and Review.

[CR28] van Ravenzwaaij D, Oberauer K (2009). How to use the diffusion model: Parameter recovery of three methods: EZ, fast-dm, and DMAT. Journal of Mathematical Psychology.

[CR29] Voss A, Rothermund K, Voss J (2004). Interpreting the parameters of the diffusion model: An empirical validation. Memory & Cognition.

[CR30] Voss A, Voss J (2007). Fast-dm: A free program for efficient diffusion model analysis. Behavior Research Methods.

[CR31] Wagenmakers E-J, van der Maas HLJ, Dolan CV, Grasman RPPP (2008). EZ Does it! Extensions of the EZ-diffusion model. Psychonomic Bulletin & Review.

[CR32] Wagenmakers E-J, van der Maas HLJ, Grasman RPPP (2007). An EZ-diffusion model for response time and accuracy. Psychonomic Bulletin & Review.

[CR33] White CN, Poldrack RA (2014). Decomposing bias in different types of simple decisions. Journal of Experimental Psychology: Learning, Memory, and Cognition.

[CR34] Wiecki, T.V., Sofer, I., & Frank, M.J. (2013). HDDM: Hierarchical Bayesian Estimation of the Drift-Diffusion Model in Python. frontiers in Neuroinformatics, 7, Article 14. 10.3389/fninf.2013.00014. 10.3389/fninf.2013.00014 PMC3731670 23935581

[CR35] Woltz DJ, Was CA (2006). Availability of related long-term memory during and after attention focus in working memory. Memory & Cognition.

