# Supplementary material for: Problems when fixing the response bias parameter z in drift diffusion analysis: A Commentary on Stafford et al. (2020)
Source: Behav Res Methods. 2022 Mar 22;55(1):168–75. doi: 10.3758/s13428-021-01786-0 (PMC9918591; doi:10.3758/s13428-021-01786-0)
Supplement: Supplementary file 1 — (PDF 1.29 MB) [file 13428_2021_1786_MOESM1_ESM.pdf]

Problems When Fixing the Response Bias Parameter z in Drift Diffusion Analysis

A Commentary on Stafford et al. (2020)

Supplemental Material

Rainer W. Alexandrowicz & Bartosz Gula

Contents

1. Introduction **2**

2. Method **2**

3. Descriptive Statistics of the Simulated Data **3**

3.1. Upper Boundary Hit Probabilities . . . 3
3.2. Total RT Means . . . 6
3.3. Upper and Lower RT Means . . . 9

4. Distributions of the Parameter Estimates **12**

4.1. Marginal Distributions . . . 12
4.2. Distribution of $\hat{z}$ . . . 15
4.3. Marginal Distributions Split by z . . . 17

5. Interactions of Parameter Estimates **18**

5.1. The Boundary Separation Parameter a . . . 18
5.1.1. ML free . . . 18
5.1.2. ML fix . . . 19
5.1.3. EZ . . . 20
5.2. The Drift Parameter ν . . . 21
5.2.1. ML free . . . 21
5.2.2. ML fix . . . 22
5.2.3. EZ . . . 23
5.3. The Encoding and Reaction Time t_{ER} . . . 24
5.3.1. ML free . . . 24
5.3.2. ML fix . . . 25
5.3.3. EZ . . . 26

A. The EZ Function **27**

References **28**

1. Introduction

This supplement presents in detail the simulation study demonstrating how fixing the response bias parameter z at 0.5 in an ML-based estimation routine or implying $z = 0.5$ by design in the EZ-method affects the estimates of the boundary separation parameter $\hat{a}$, the drift parameter $\hat{\nu}$, and the encoding and response time $\hat{t}_{\text{ER}}$.

The starting point/response bias parameter z may be expressed in two ways, either absolute (i. e., $0 < z_{\text{abs}} < a$) or relative to a (i. e., $0 < z_{\text{rel}} < 1$; $z_{\text{abs}} = a \cdot z_{\text{rel}}$). We will refer to z_{rel} throughout this text.

2. Method

The simulation comprised a full grid search across the model parameter values given in Table 1.

Table 1: Simulation Parameters

Parameter	Values
a	1, 2, 3
z_{rel}	0.3, 0.5, 0.7
ν	–2, –1, 0, 1, 2
t_{ER}	0.1, 0.3, 0.5, 0.7, 1

The parameter values were chosen to cover a wide range of possible parameter combinations to explore in detail the impact of fixing $z = 0.5$ under a great variety of outcomes. Not all of these combinations are likely to appear in applications, but the approach allows for detecting the systematics of effects.

We chose $k = 400$ trials and 20 replications per parameter combination, as these values were found to provide sufficiently stable results in a series of preliminary simulation runs. This yields a total of $3a \times 5\nu \times 3z \times 5t_{\text{ER}} \times 20 = 4{,}500$ samples.

All calculations were performed with R (R Core Team, 2020), using the `rdiffusion()` function from the `rtdists` package (Singmann, Brown, Gretton, & Heathcote, 2020) for data simulation and the `wdm()` function from the `RWiener` package (Wabersich & Vandekerckhove, 2014) for the ML-based parameter estimation. The EZ algorithm was implemented according to the routine provided by Wagenmakers, van der Maas, and Grasman (2007, see Appendix A).

3. Descriptive Statistics of the Simulated Data

Before analysing in detail the various parameters' estimates, we check the success of the simulation by inspecting the sample descriptives predictable from the model equations.

3.1. Upper Boundary Hit Probabilities

According to Ratcliff (1978, Eq. (1), p. 70), the Drift Diffusion Model defines the probability of a lower boundary hit as

$$P(-|a, z, \nu) = \frac{e^{-2va/s^2} - e^{-2vz/s^2}}{e^{-2va/s^2} - 1} \tag{1}$$

(notation adapted; note that Equation (1) refers to $z_{\text{abs}} = z_{\text{rel}} \cdot a$). With this equation, we may calculate the expected probabilities of either boundary hit from the simulation parameters and compare them to the observed means (Figure 1, showing $P(+)$ here).

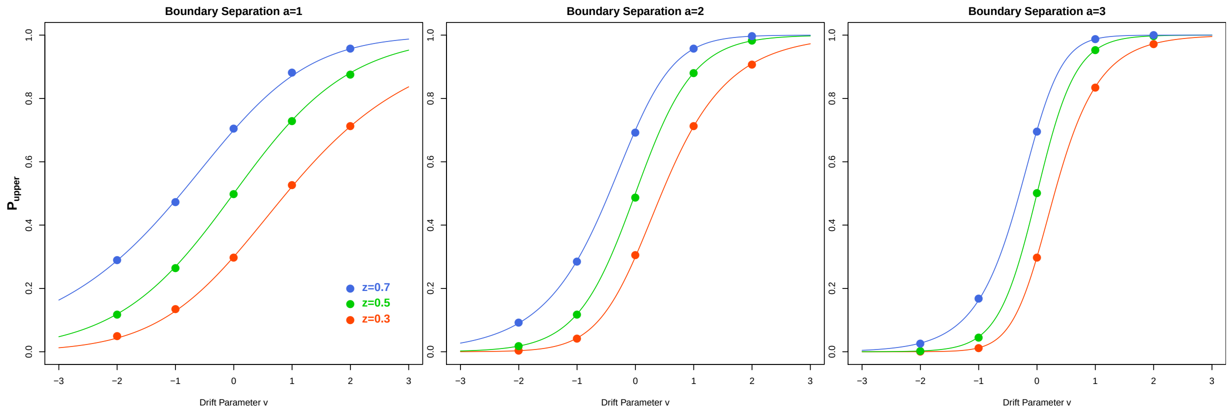

Figure 1: Expected (lines) and observed means (bullets) of upper hit probabilities.

The lines in Figure 1 show the expected means of upper hit probabilities according to Equation (1) for ν ranging from -3 to 3. The bullets indicate the means of the replicated data sets with the respective combinations of a, ν, and z_{rel}. We find virtually perfect agreement of observed and expected proportions of upper threshold hits across all combinations of a, ν, and z, as the bullets are located exactly on the respective lines. The differences of observed and expected do not exceed ± 0.08 (for a complete breakdown by the levels of a, z_{rel}, and ν see Table 2).

Table 2: Deviations of observed $P(+)$ from expected.

a	z_{rel}	ν	Min.	1st Qu.	Median	3rd Qu.	Max.	Mean	SD
1	0.3	−2	−0.028	−0.003	0.007	0.014	0.024	0.005	0.010
2	0.3	−2	−0.003	−0.001	−0.001	0.002	0.007	0.000	0.003
3	0.3	−2	−0.000	−0.000	−0.000	−0.000	0.002	0.000	0.001
1	0.5	−2	−0.044	−0.019	−0.000	0.009	0.043	−0.002	0.022
2	0.5	−2	−0.013	−0.005	−0.000	0.005	0.012	−0.000	0.006
3	0.5	−2	−0.002	−0.002	0.000	0.000	0.005	−0.000	0.002
1	0.7	−2	−0.043	−0.016	−0.001	0.012	0.079	0.002	0.026
2	0.7	−2	−0.028	−0.006	0.005	0.012	0.035	0.002	0.014
3	0.7	−2	−0.015	−0.005	−0.002	0.003	0.013	−0.002	0.007
1	0.3	−1	−0.026	−0.006	0.004	0.016	0.044	0.005	0.015
2	0.3	−1	−0.031	−0.011	−0.003	0.007	0.024	−0.001	0.012
3	0.3	−1	−0.010	−0.005	−0.000	0.002	0.007	−0.000	0.004
1	0.5	−1	−0.059	−0.019	−0.004	0.014	0.064	−0.005	0.026
2	0.5	−1	−0.027	−0.012	0.001	0.006	0.031	−0.001	0.013
3	0.5	−1	−0.017	−0.012	−0.005	0.005	0.028	−0.003	0.011
1	0.7	−1	−0.041	−0.019	−0.003	0.012	0.069	−0.005	0.021
2	0.7	−1	−0.046	−0.023	−0.003	0.017	0.049	−0.003	0.022
3	0.7	−1	−0.056	−0.006	0.002	0.019	0.049	0.004	0.017
1	0.3	0	−0.085	−0.016	−0.003	0.020	0.050	−0.002	0.028
2	0.3	0	−0.038	−0.015	0.002	0.027	0.037	0.005	0.022
3	0.3	0	−0.055	−0.015	−0.001	0.013	0.050	−0.003	0.022
1	0.5	0	−0.048	−0.023	0.005	0.011	0.045	−0.002	0.023
2	0.5	0	−0.063	−0.033	−0.016	0.002	0.055	−0.013	0.030
3	0.5	0	−0.055	−0.015	0.007	0.012	0.040	0.000	0.026
1	0.7	0	−0.033	−0.010	0.004	0.022	0.045	0.005	0.023
2	0.7	0	−0.058	−0.020	−0.013	0.007	0.045	−0.008	0.021
3	0.7	0	−0.045	−0.013	−0.005	0.003	0.025	−0.005	0.014
1	0.3	1	−0.047	−0.009	0.006	0.018	0.048	0.005	0.023
2	0.3	1	−0.032	−0.019	0.001	0.017	0.043	0.000	0.021
3	0.3	1	−0.037	−0.019	−0.002	0.011	0.036	−0.003	0.019
1	0.5	1	−0.046	−0.024	−0.004	0.014	0.041	−0.003	0.023
2	0.5	1	−0.036	−0.011	0.002	0.012	0.027	−0.000	0.016
3	0.5	1	−0.020	−0.005	−0.003	0.007	0.035	−0.001	0.011
1	0.7	1	−0.019	−0.001	0.011	0.016	0.046	0.010	0.016
2	0.7	1	−0.022	−0.007	0.003	0.008	0.023	0.001	0.011
3	0.7	1	−0.012	−0.005	0.003	0.003	0.010	0.000	0.006
1	0.3	2	−0.057	−0.014	0.001	0.018	0.043	0.001	0.021
2	0.3	2	−0.032	−0.012	−0.002	0.008	0.023	−0.003	0.014
3	0.3	2	−0.023	−0.008	−0.000	0.005	0.015	−0.002	0.009
1	0.5	2	−0.043	−0.016	−0.003	0.007	0.029	−0.005	0.018
2	0.5	2	−0.015	−0.005	0.003	0.005	0.013	0.001	0.007
3	0.5	2	−0.005	−0.003	−0.000	0.002	0.002	−0.000	0.002
1	0.7	2	−0.024	−0.005	0.001	0.008	0.021	0.001	0.010
2	0.7	2	−0.012	−0.004	0.001	0.001	0.003	−0.000	0.003
3	0.7	2	−0.002	0.000	0.000	0.000	0.000	0.000	0.000

Figure 2 shows how $P(+)$ is generally associated with a, ν, and z.

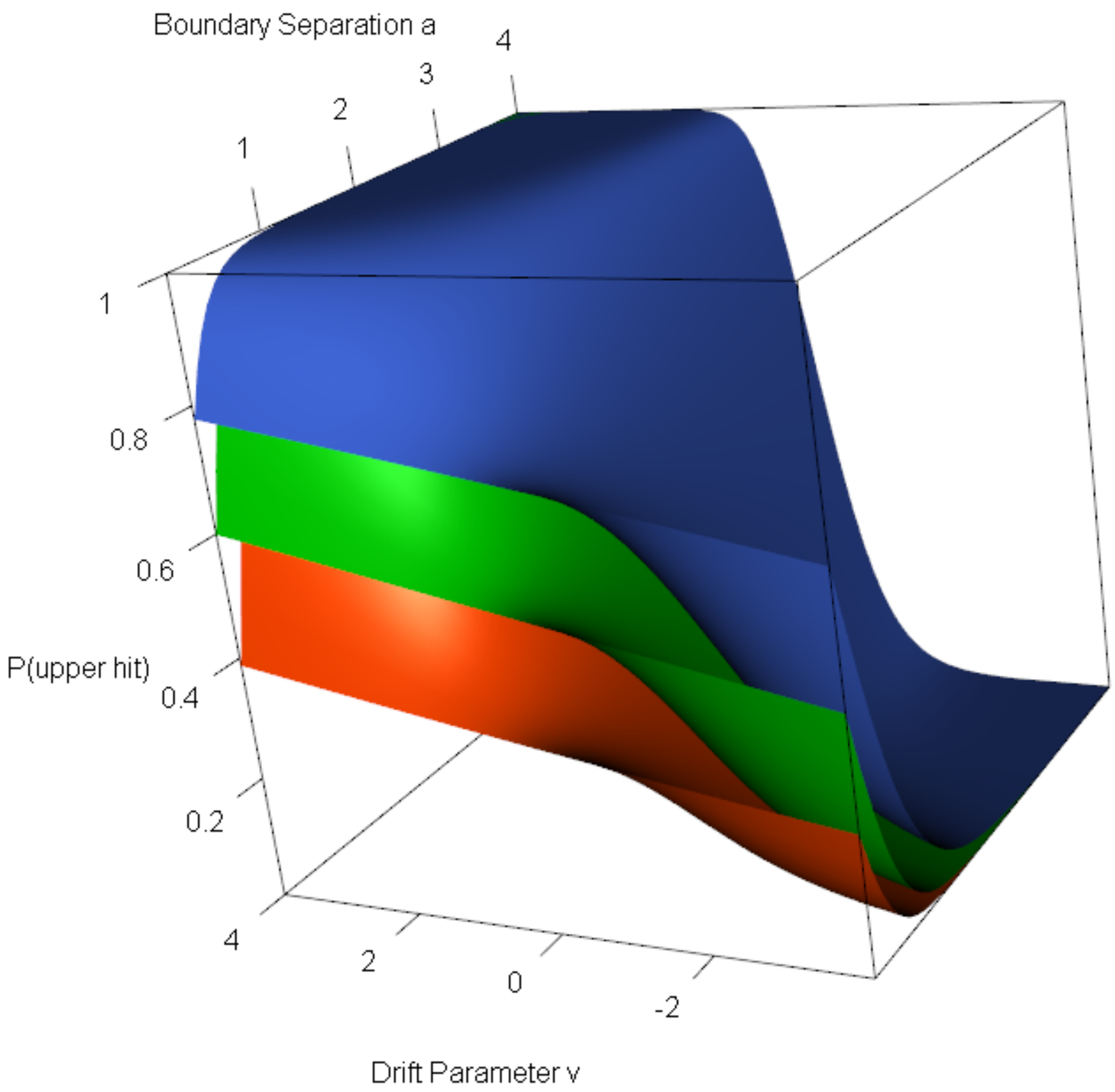

Figure 2: $P(+)$ (vertical) by a and ν (axes), and z_{rel} (layers, for color codes see Figure 1).

3.2. Total RT Means

Grasman, Wagenmakers, and van der Maas (2009) give a closed form expression for the expected mean of the total response times (i. e., combined upper and lower boundary hits):

$$E(T) = -\frac{z}{\nu} + \frac{a}{\nu} \cdot \frac{e^{-2\nu z/s^2} - 1}{e^{-2\nu a/s^2} - 1} \tag{2}$$

(ibid., Equation (5), p. 58). Figure 3 shows the observed and expected total response time means of the simulation.

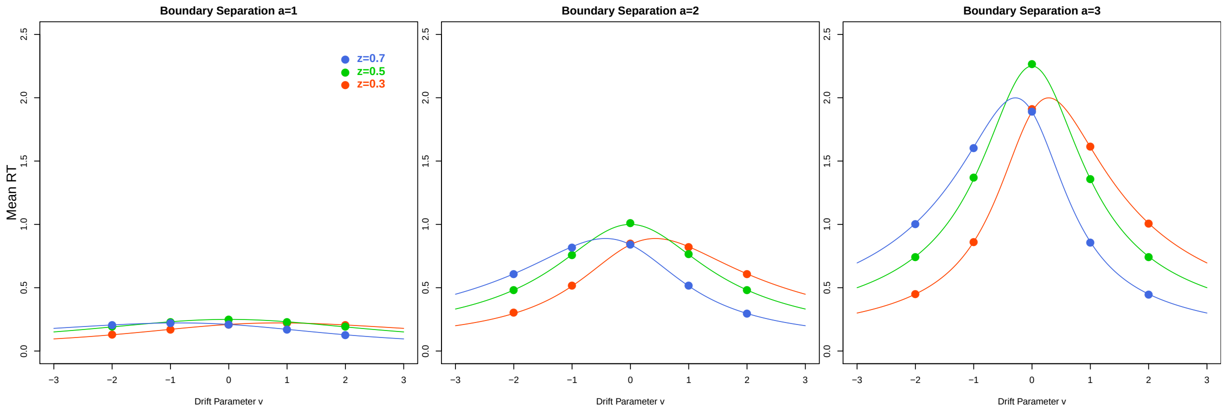

Figure 3: Expected (lines) and observed (bullets) means of total response time. For color codes see Figure 1.

Again, we find almost perfect agreement of observed and expected RT means across all combinations of a, ν, and z. The differences of the expected and the observed means range from -0.19 s to $+0.24$ s (for a complete breakdown by the levels of a, z_{rel}, and ν see Table 3).

Table 3: Deviations of observed RT means from expected.

a	z_{rel}	ν	Min.	1st Qu.	Median	3rd Qu.	Max.	Mean	SD
1	0.3	–2	–0.012	–0.003	0.001	0.004	0.015	0.000	0.005
2	0.3	–2	–0.028	–0.002	0.006	0.019	0.032	0.005	0.015
3	0.3	–2	–0.034	–0.010	0.005	0.008	0.039	0.002	0.016
1	0.5	–2	–0.012	–0.004	0.000	0.005	0.015	0.000	0.006
2	0.5	–2	–0.038	–0.012	–0.003	0.004	0.035	–0.001	0.016
3	0.5	–2	–0.050	–0.023	–0.012	0.017	0.055	–0.005	0.026
1	0.7	–2	–0.020	–0.003	–0.001	0.006	0.014	0.001	0.008
2	0.7	–2	–0.039	–0.023	–0.001	0.012	0.036	–0.003	0.020
3	0.7	–2	–0.048	–0.027	–0.014	0.014	0.058	–0.008	0.027
1	0.3	–1	–0.022	–0.010	0.000	0.005	0.014	–0.002	0.010
2	0.3	–1	–0.032	–0.016	–0.001	0.015	0.062	0.004	0.025
3	0.3	–1	–0.130	–0.026	–0.002	0.023	0.085	–0.003	0.041
1	0.5	–1	–0.025	–0.008	–0.001	0.006	0.017	–0.001	0.011
2	0.5	–1	–0.065	–0.034	–0.014	0.032	0.060	–0.005	0.036
3	0.5	–1	–0.081	–0.024	0.006	0.043	0.197	0.012	0.060
1	0.7	–1	–0.023	–0.005	–0.000	0.007	0.019	0.001	0.010
2	0.7	–1	–0.059	–0.031	–0.007	0.013	0.053	–0.008	0.029
3	0.7	–1	–0.083	–0.031	–0.020	0.022	0.078	–0.008	0.043
1	0.3	0	–0.025	–0.006	–0.000	0.007	0.032	–0.000	0.012
2	0.3	0	–0.080	–0.029	0.010	0.038	0.091	0.008	0.040
3	0.3	0	–0.194	–0.046	0.010	0.087	0.242	0.022	0.100
1	0.5	0	–0.030	–0.008	0.002	0.005	0.025	–0.002	0.012
2	0.5	0	–0.101	–0.023	0.004	0.038	0.099	0.011	0.045
3	0.5	0	–0.151	–0.053	0.029	0.073	0.202	0.016	0.091
1	0.7	0	–0.021	–0.010	–0.003	0.008	0.020	–0.001	0.011
2	0.7	0	–0.088	–0.025	0.003	0.032	0.070	–0.000	0.043
3	0.7	0	–0.138	–0.056	–0.006	0.046	0.239	–0.001	0.071
1	0.3	1	–0.021	–0.010	–0.003	0.008	0.019	–0.001	0.011
2	0.3	1	–0.066	–0.016	0.001	0.019	0.054	–0.002	0.029
3	0.3	1	–0.101	–0.036	0.002	0.039	0.134	0.004	0.058
1	0.5	1	–0.022	–0.008	–0.003	0.004	0.014	–0.003	0.009
2	0.5	1	–0.061	–0.012	0.003	0.029	0.047	0.005	0.027
3	0.5	1	–0.119	–0.032	0.007	0.032	0.115	–0.002	0.052
1	0.7	1	–0.021	–0.004	–0.001	0.005	0.021	–0.000	0.009
2	0.7	1	–0.043	–0.012	0.004	0.017	0.061	0.005	0.023
3	0.7	1	–0.098	–0.042	0.001	0.028	0.069	–0.006	0.048
1	0.3	2	–0.018	–0.003	–0.001	0.004	0.010	–0.000	0.006
2	0.3	2	–0.038	–0.016	–0.002	0.007	0.033	–0.003	0.017
3	0.3	2	–0.055	–0.021	–0.005	0.011	0.057	–0.004	0.025
1	0.5	2	–0.021	–0.001	0.002	0.005	0.013	0.001	0.007
2	0.5	2	–0.040	–0.011	–0.002	0.010	0.040	–0.001	0.017
3	0.5	2	–0.039	–0.012	–0.005	0.001	0.044	–0.006	0.014
1	0.7	2	–0.017	–0.004	–0.001	0.005	0.014	–0.001	0.007
2	0.7	2	–0.022	–0.011	–0.000	0.011	0.023	–0.001	0.013
3	0.7	2	–0.028	–0.016	–0.005	0.002	0.026	–0.005	0.012

Figure 4 shows the expected means of the total RTs (i. e., upper and lower combined) by boundary separation a and drift parameter ν.

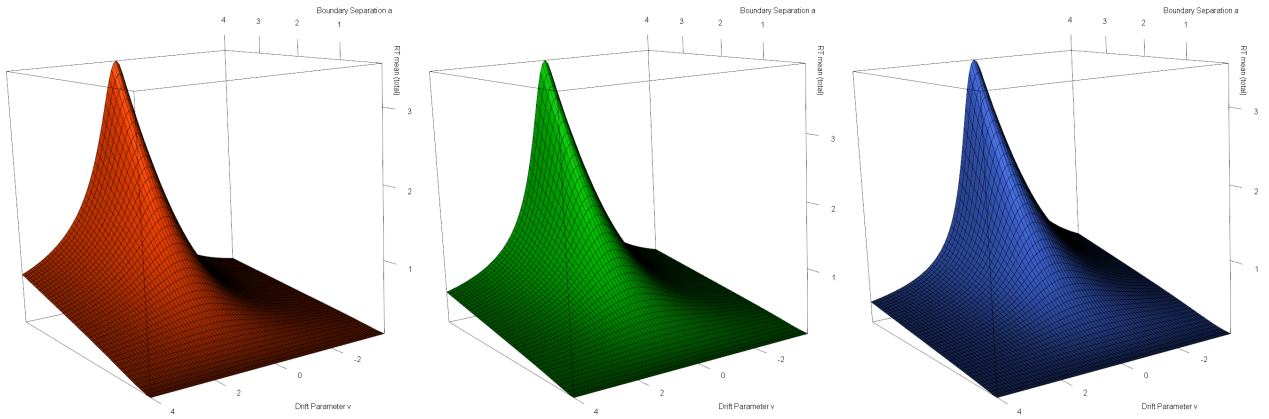

(a) $z_{\text{rel}} = 0.3$ (b) $z_{\text{rel}} = 0.5$ (c) $z_{\text{rel}} = 0.7$

Figure 4: Expected RT means by $a \times \nu$ for three levels of z.

3.3. Upper and Lower RT Means

Grasman et al. (2009) also provided closed form expressions for the expected means of upper and lower threshold RTs by using the auxiliary function

$$\varphi(x, y) = \mathrm{e}^{2\nu y/s^2} - \mathrm{e}^{2\nu x/s^2} \quad (3)$$

to express the expected mean of the lower threshold hits by

$$E(RT_{\text{lower}}) = \frac{z(\varphi(z - a, a) + \varphi(0, z) + 2a\varphi(z, 0)}{\nu\varphi(z, a)\varphi(-a, 0)} \quad (4)$$

(ibid., Equation (13), p. 59) and $E(RT_{\text{upper}})$ by transforming $\nu \mapsto -\nu$ and $z \mapsto a - z$ (again referring to z_{abs}). Figure 5 shows the expected vs. the observed means for the upper and the lower threshold hits.

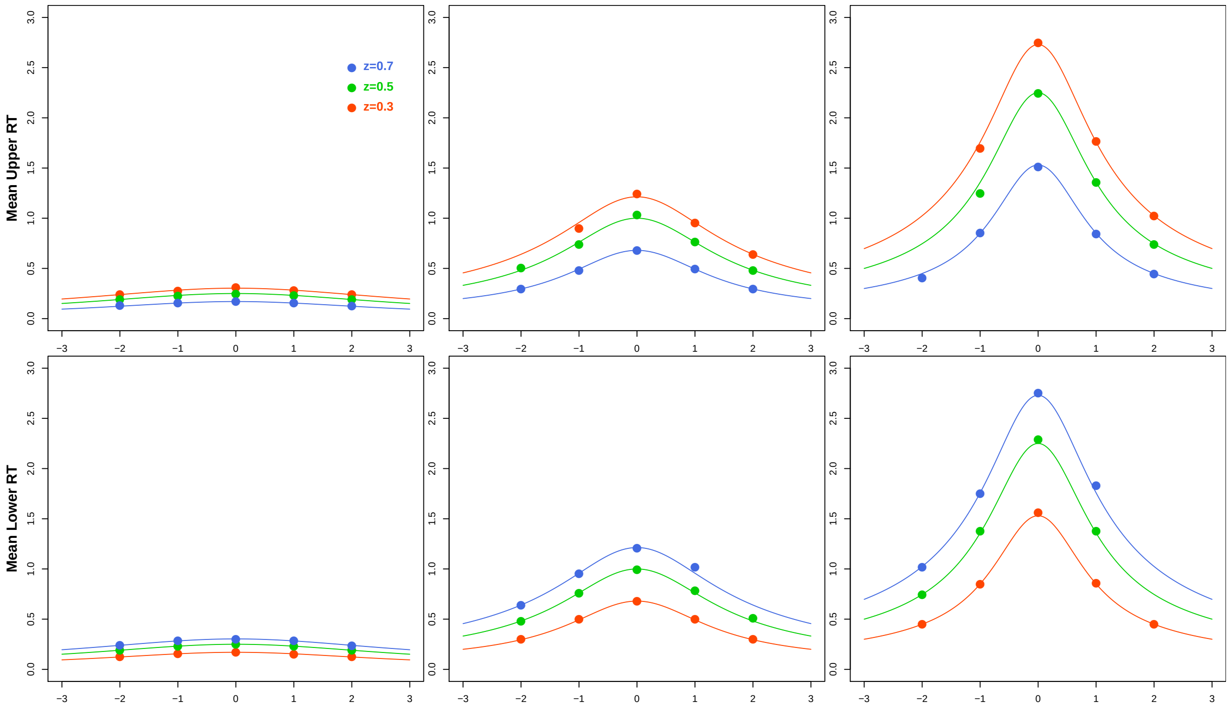

Figure 5: Observed (bullets) and expected (lines) means of upper and lower RT distributions. Notes: Horizontal axis: $\nu = -3 \ldots + 3$; Left column: $a = 1$, middle column: $a = 2$, and right column: $a = 3$.

Also the means of the upper and the lower distribution provide excellent agreement with their expected values across the various parameter combinations (both $r = 0.98$). However, due to smaller subsample sizes, some means deviate slightly more than in the total sample, and a few means (bullets) are missing, because in some samples with extreme parameter combinations, only one boundary was hit. However, differences still appear very acceptable (see Tables 4 and 5 for descriptive statistics of deviations).

Table 4: Deviations of observed upper RT means from expected.

a	z_{rel}	ν	Min.	1st Qu.	Median	3rd Qu.	Max.	Mean	SD
1	0.3	–2	–0.084	–0.020	0.001	0.019	0.148	0.000	0.036
2	0.3	–2	–0.477	–0.231	0.085	0.261	1.142	0.086	0.386
3	0.3	–2	–0.405	–0.405	0.514	0.514	0.671	0.123	0.503
1	0.5	–2	–0.038	–0.015	–0.002	0.010	0.083	–0.003	0.018
2	0.5	–2	–0.245	–0.052	0.021	0.098	0.297	0.023	0.126
3	0.5	–2	–0.553	–0.278	–0.130	0.035	0.820	–0.098	0.293
1	0.7	–2	–0.026	–0.003	0.004	0.012	0.042	0.005	0.017
2	0.7	–2	–0.095	–0.017	0.001	0.013	0.100	–0.003	0.040
3	0.7	–2	–0.214	–0.115	–0.094	0.033	0.291	–0.047	0.114
1	0.3	–1	–0.059	–0.024	–0.012	0.003	0.066	–0.011	0.025
2	0.3	–1	–0.336	–0.133	–0.059	0.012	0.397	–0.061	0.126
3	0.3	–1	–0.965	–0.317	–0.146	0.093	1.109	–0.062	0.434
1	0.5	–1	–0.074	–0.020	–0.003	0.011	0.045	–0.005	0.023
2	0.5	–1	–0.148	–0.098	–0.028	0.029	0.208	–0.022	0.090
3	0.5	–1	–0.440	–0.235	–0.125	0.029	0.289	–0.112	0.192
1	0.7	–1	–0.026	–0.008	–0.001	0.009	0.024	–0.000	0.012
2	0.7	–1	–0.104	–0.044	–0.008	0.015	0.125	–0.013	0.040
3	0.7	–1	–0.163	–0.069	–0.001	0.068	0.251	0.002	0.090
1	0.3	0	–0.024	–0.011	0.011	0.014	0.049	0.004	0.019
2	0.3	0	–0.153	0.001	0.048	0.074	0.145	0.028	0.080
3	0.3	0	–0.309	–0.136	0.041	0.129	0.458	0.018	0.185
1	0.5	0	–0.026	–0.013	–0.004	0.011	0.030	–0.003	0.015
2	0.5	0	–0.122	–0.002	0.013	0.089	0.204	0.033	0.069
3	0.5	0	–0.246	–0.120	–0.007	0.100	0.326	–0.004	0.138
1	0.7	0	–0.018	–0.008	–0.001	0.012	0.022	0.001	0.011
2	0.7	0	–0.113	–0.035	–0.007	0.036	0.104	–0.004	0.046
3	0.7	0	–0.203	–0.062	–0.016	0.028	0.148	–0.019	0.081
1	0.3	1	–0.028	–0.007	0.000	0.007	0.027	–0.001	0.012
2	0.3	1	–0.074	–0.032	0.001	0.023	0.082	–0.004	0.039
3	0.3	1	–0.116	–0.057	0.009	0.045	0.125	0.007	0.060
1	0.5	1	–0.026	–0.008	–0.005	0.004	0.031	–0.003	0.012
2	0.5	1	–0.076	–0.017	–0.000	0.031	0.061	0.003	0.034
3	0.5	1	–0.098	–0.039	0.005	0.033	0.120	–0.003	0.050
1	0.7	1	–0.020	–0.004	0.000	0.007	0.017	0.001	0.009
2	0.7	1	–0.052	–0.010	–0.001	0.016	0.065	0.003	0.025
3	0.7	1	–0.110	–0.046	–0.000	0.030	0.064	–0.006	0.049
1	0.3	2	–0.015	–0.006	–0.000	0.003	0.016	–0.001	0.006
2	0.3	2	–0.041	–0.020	–0.003	0.015	0.038	–0.002	0.020
3	0.3	2	–0.059	–0.019	–0.005	0.009	0.061	–0.004	0.024
1	0.5	2	–0.022	–0.000	0.003	0.006	0.013	0.001	0.007
2	0.5	2	–0.040	–0.012	–0.004	0.009	0.038	–0.001	0.017
3	0.5	2	–0.037	–0.012	–0.006	0.002	0.044	–0.006	0.014
1	0.7	2	–0.018	–0.003	–0.000	0.003	0.015	–0.000	0.007
2	0.7	2	–0.026	–0.012	0.000	0.012	0.023	–0.001	0.013
3	0.7	2	–0.028	–0.016	–0.004	0.002	0.026	–0.005	0.012

Table 5: Deviations of observed lower RT means from expected.

a	z_{rel}	ν	Min.	1st Qu.	Median	3rd Qu.	Max.	Mean	SD
1	0.3	–2	–0.012	–0.004	–0.000	0.003	0.016	–0.000	0.005
2	0.3	–2	–0.027	–0.003	0.004	0.017	0.034	0.005	0.015
3	0.3	–2	–0.033	–0.010	0.004	0.008	0.039	0.002	0.016
1	0.5	–2	–0.013	–0.005	0.000	0.004	0.019	0.001	0.007
2	0.5	–2	–0.038	–0.013	–0.003	0.006	0.034	–0.002	0.016
3	0.5	–2	–0.050	–0.023	–0.012	0.017	0.055	–0.005	0.026
1	0.7	–2	–0.020	–0.005	–0.001	0.006	0.014	–0.001	0.008
2	0.7	–2	–0.036	–0.022	–0.002	0.015	0.035	–0.003	0.020
3	0.7	–2	–0.050	–0.030	–0.010	0.012	0.059	–0.008	0.027
1	0.3	–1	–0.019	–0.011	–0.000	0.004	0.015	–0.001	0.009
2	0.3	–1	–0.039	–0.008	0.004	0.020	0.068	0.007	0.024
3	0.3	–1	–0.134	–0.030	0.005	0.027	0.095	–0.001	0.043
1	0.5	–1	–0.028	–0.006	–0.001	0.008	0.024	0.000	0.011
2	0.5	–1	–0.070	–0.023	–0.012	0.032	0.057	–0.003	0.036
3	0.5	–1	–0.088	–0.015	0.012	0.047	0.209	0.018	0.062
1	0.7	–1	–0.026	–0.012	0.000	0.008	0.031	0.000	0.013
2	0.7	–1	–0.082	–0.045	–0.002	0.013	0.094	–0.007	0.041
3	0.7	–1	–0.085	–0.045	–0.008	0.029	0.119	–0.006	0.045
1	0.3	0	–0.023	–0.008	–0.000	0.005	0.026	–0.002	0.012
2	0.3	0	–0.128	–0.034	0.002	0.024	0.116	–0.004	0.048
3	0.3	0	–0.219	–0.017	0.046	0.078	0.292	0.029	0.101
1	0.5	0	–0.033	–0.007	0.001	0.006	0.021	–0.000	0.013
2	0.5	0	–0.113	–0.041	–0.017	0.007	0.124	–0.009	0.058
3	0.5	0	–0.140	–0.050	0.034	0.087	0.304	0.038	0.106
1	0.7	0	–0.039	–0.017	–0.010	0.009	0.038	–0.004	0.018
2	0.7	0	–0.164	–0.048	–0.003	0.045	0.208	–0.008	0.072
3	0.7	0	–0.373	–0.065	–0.012	0.109	0.470	0.020	0.140
1	0.3	1	–0.032	–0.012	–0.003	0.009	0.020	–0.002	0.013
2	0.3	1	–0.074	–0.031	–0.005	0.021	0.117	0.006	0.045
3	0.3	1	–0.183	–0.041	0.001	0.063	0.210	0.007	0.085
1	0.5	1	–0.031	–0.010	0.001	0.005	0.037	–0.002	0.013
2	0.5	1	–0.192	–0.029	0.011	0.061	0.201	0.021	0.081
3	0.5	1	–0.453	–0.195	0.085	0.202	0.543	0.019	0.228
1	0.7	1	–0.055	–0.024	0.005	0.017	0.065	0.002	0.030
2	0.7	1	–0.208	–0.061	0.059	0.135	0.602	0.058	0.177
3	0.7	1	–1.019	–0.317	–0.096	0.226	3.342	0.072	0.738
1	0.3	2	–0.023	–0.009	0.001	0.006	0.026	0.001	0.012
2	0.3	2	–0.078	–0.019	0.004	0.028	0.093	0.003	0.044
3	0.3	2	–0.151	–0.073	–0.039	0.093	0.270	0.001	0.106
1	0.5	2	–0.048	–0.015	–0.000	0.013	0.041	–0.002	0.020
2	0.5	2	–0.225	–0.096	0.045	0.104	0.465	0.028	0.139
3	0.5	2	–0.509	–0.284	–0.122	0.126	0.948	–0.042	0.361
1	0.7	2	–0.090	–0.035	–0.009	0.021	0.094	–0.004	0.038
2	0.7	2	–0.396	–0.109	0.024	0.181	0.472	0.019	0.213
3	0.7	2	–0.452	–0.177	0.098	0.373	0.648	0.098	0.778

4. Distributions of the Parameter Estimates

After ascertaining that the simulated data are in excellent agreement with the expected distributions according to the model and the true parameters, we now focus on the parameter estimates compared across the three estimation methods.

4.1. Marginal Distributions

Figure 6 shows the marginal distributions of a, ν, and t_{ER} for the three estimation methods free ML, ML with z fixed at 0.5, and the EZ algorithm.

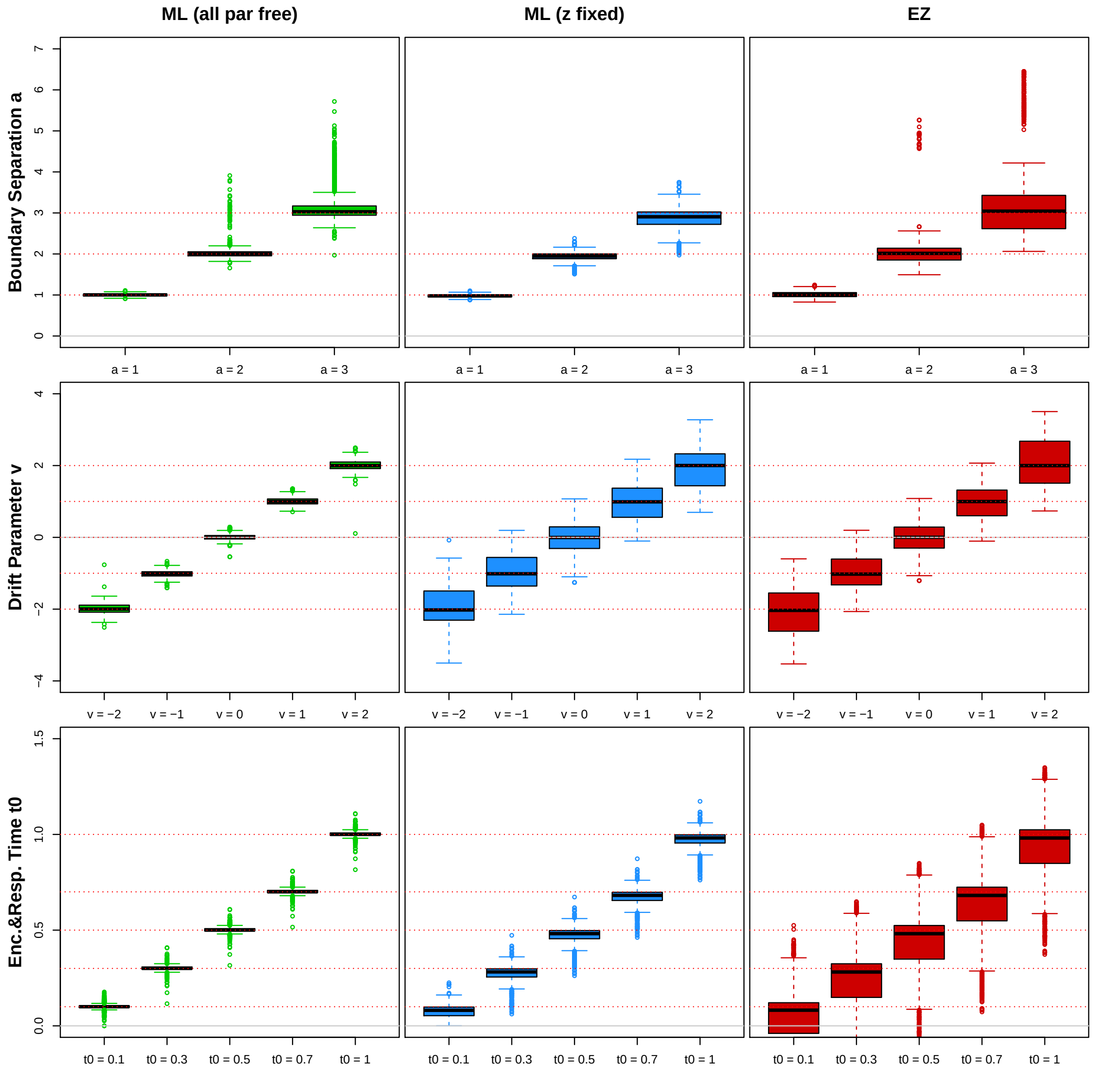

Figure 6: Parameter recovery (marginal distributions).

First and foremost, the ML free estimates (green, left column) of a, ν, and t_{ER} agree excellently with the population values and exhibit also low variability (i. e., standard error). However, we find a few outliers for a (top left diagram of Figure 6), which are accounted for by parameter combinations yielding hit probabilities of (almost) zero or one (Figure 7).

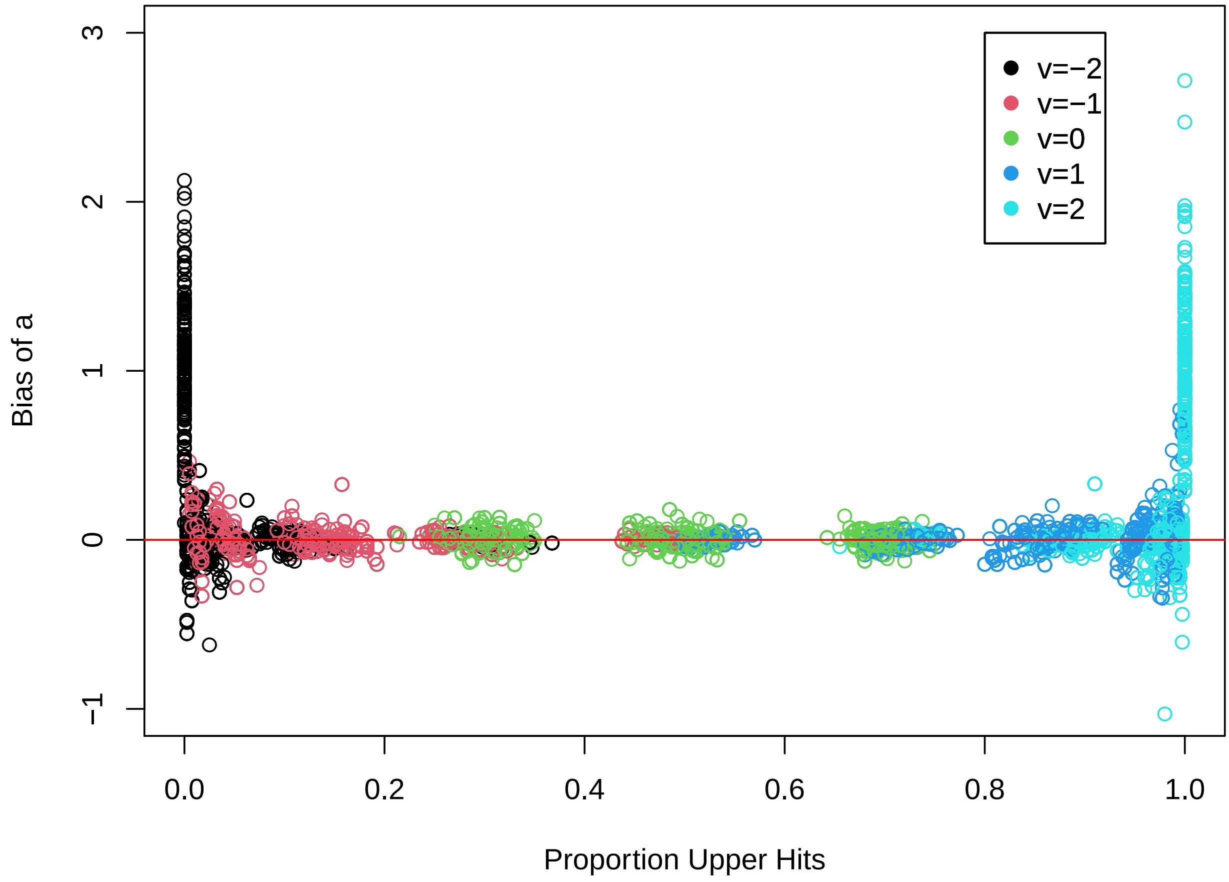

Figure 7: Bias of $\hat{a}$ (vertical) by upper hit proportions.

Also for the ML method with z fixed at zero (blue, middle column in Figure 6), we find barely a bias, yet a larger variability of the drift parameter ν, which will be explored further in Section 5.2.2.

The estimates obtained with EZ method (red, right column in Figure 6) show larger variability of all three parameters, but – most disturbingly – the estimates of t_{ER} fall short of zero (for true values smaller or equal 0.3), which do not constitute valid estimates at all. Figure 8 shows the entire distribution of the t_{ER}, where we find negative outliers up to almost –2.

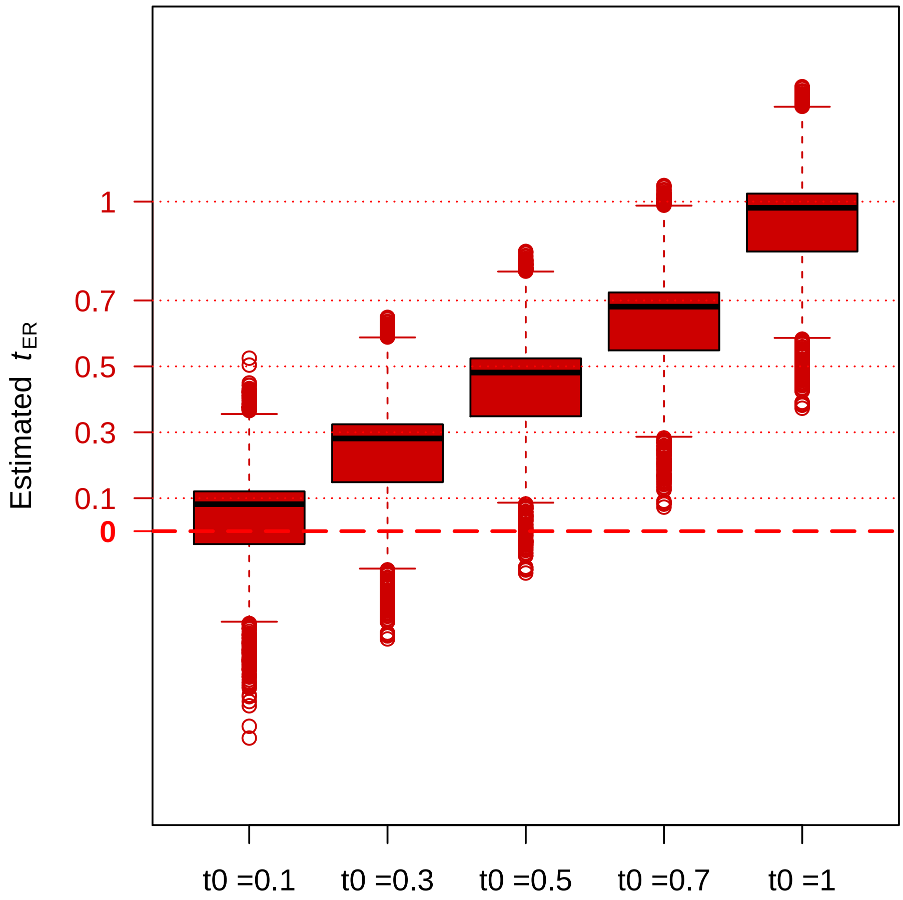

Figure 8: EZ estimates of t_{ER}.

We, therefore, may already conclude from this marginal evaluation that EZ does not allow for a reasonable estimate of the t_{ER} (at least for small encoding and reaction times).

4.2. Distribution of $\hat{z}$

The bias parameter z was only estimated by the `ML free` method (Figure 9).

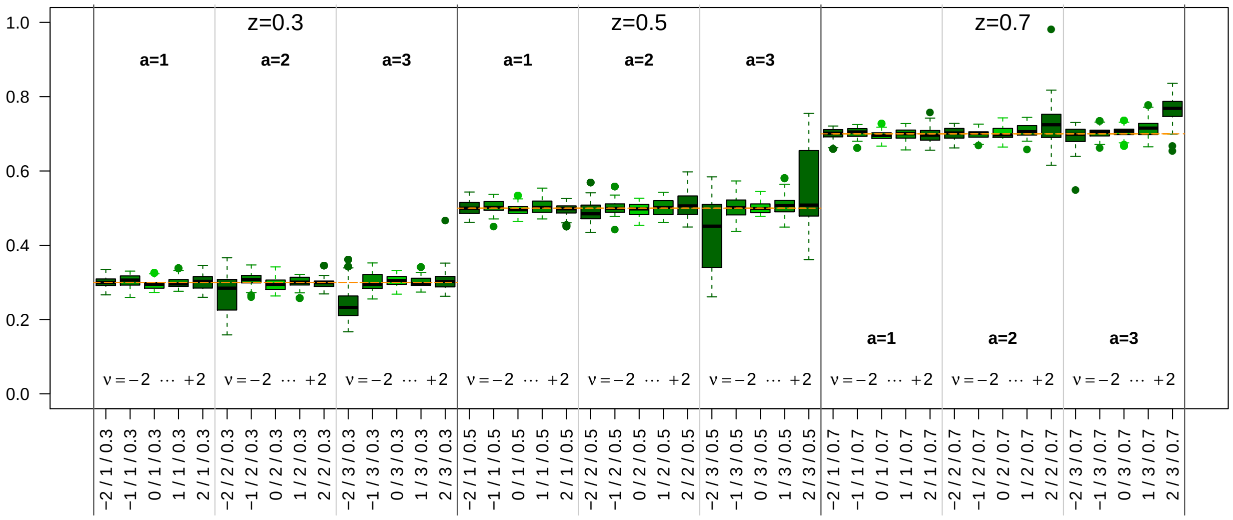

Figure 9: Estimates $\hat{z}_{\text{ML free}}$ by $\nu \times a \times z$

Generally, there is no systematic bias of $\hat{z}$ discernible, however, some parameter combinations involving large $|\nu|$ indeed exhibit deviating distributions (Table 6).

Table 6: Means (M) and standard deviations (SD) of $\hat{z}$ by $z \times a \times \nu$.

		$z = 0.3$			$z = 0.5$			$z = 0.7$		
a		1	2	3	1	2	3	1	2	3
$\nu = -2$	M	0.30	0.28	0.24	0.50	0.49	0.43	0.70	0.70	0.69
	SD	0.02	0.05	0.05	0.02	0.03	0.09	0.02	0.02	0.03
$\nu = -1$	M	0.30	0.31	0.30	0.50	0.50	0.50	0.70	0.70	0.70
	SD	0.02	0.02	0.02	0.02	0.02	0.03	0.01	0.01	0.01
$\nu = 0$	M	0.29	0.29	0.30	0.50	0.49	0.50	0.70	0.70	0.70
	SD	0.01	0.02	0.02	0.02	0.02	0.02	0.01	0.02	0.02
$\nu = +1$	M	0.30	0.30	0.30	0.50	0.50	0.50	0.70	0.71	0.71
	SD	0.01	0.02	0.01	0.02	0.02	0.03	0.02	0.02	0.03
$\nu = +2$	M	0.30	0.30	0.31	0.49	0.51	0.55	0.70	0.72	0.77
	SD	0.02	0.02	0.03	0.02	0.04	0.10	0.02	0.06	0.03

Figure 10 shows the bias of the $\hat{z}$ by the proportion of upper hit, $\hat{P}(+)$.

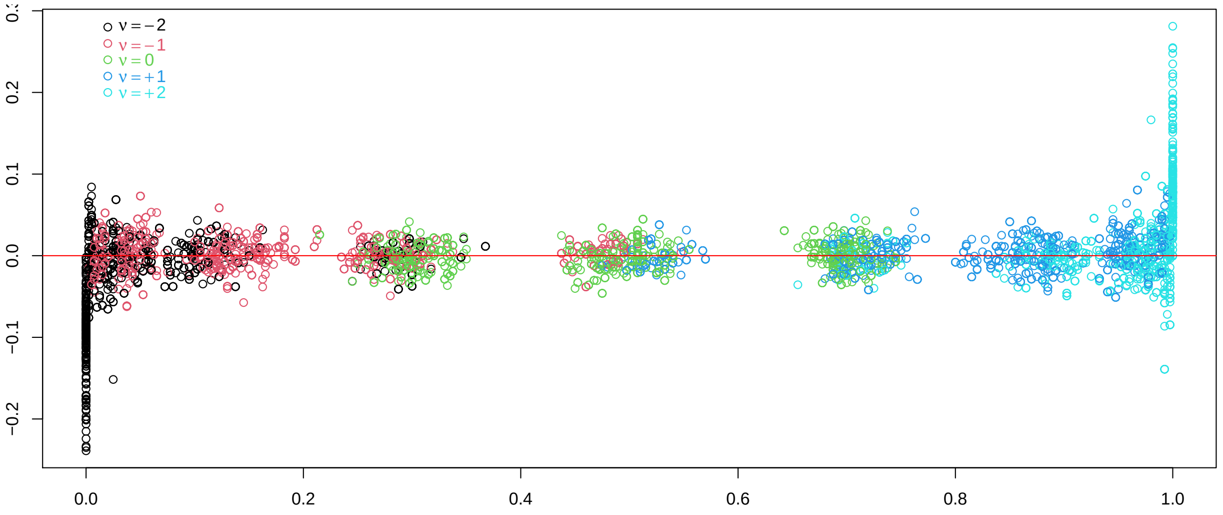

Figure 10: Bias of $\hat{z}$ by $\hat{P}(+)$.

Clearly, the deviating $\hat{z}$ are associated with vanishing probabilities of an upper or lower boundary hit. Parameter combinations yielding such extreme probabilities have no or just few hits at the boundary with the low or vanishing hit probability so that the estimates become unstable. Such extreme cases pose general estimation problems. The bias of all other $\hat{z}$ is smaller than ± 0.06 as Figure 11 shows, which is identical to Figure 10 except that data sets with $\hat{P}(+) \leq 0.03$ and $\hat{P}(+) \geq 0.97$ were omitted.

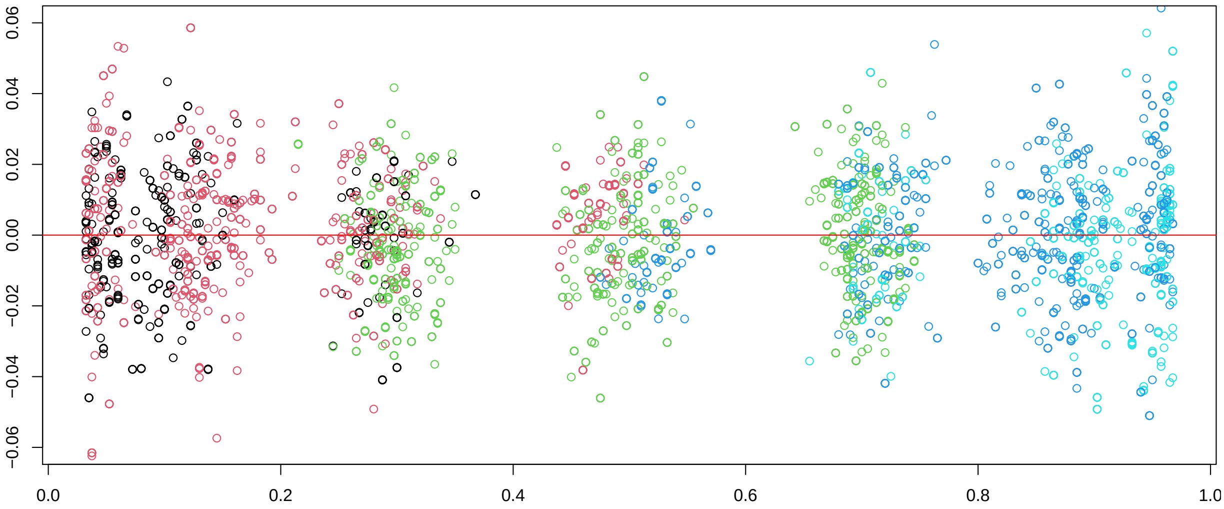

Figure 11: Bias of $\hat{z}$ by $\hat{P}^{*}(+) = 0.03 < \hat{P}(+) < 0.97$.

4.3. Marginal Distributions Split by z

By splitting the estimates' distributions by the bias parameter z, we find some irregularities:

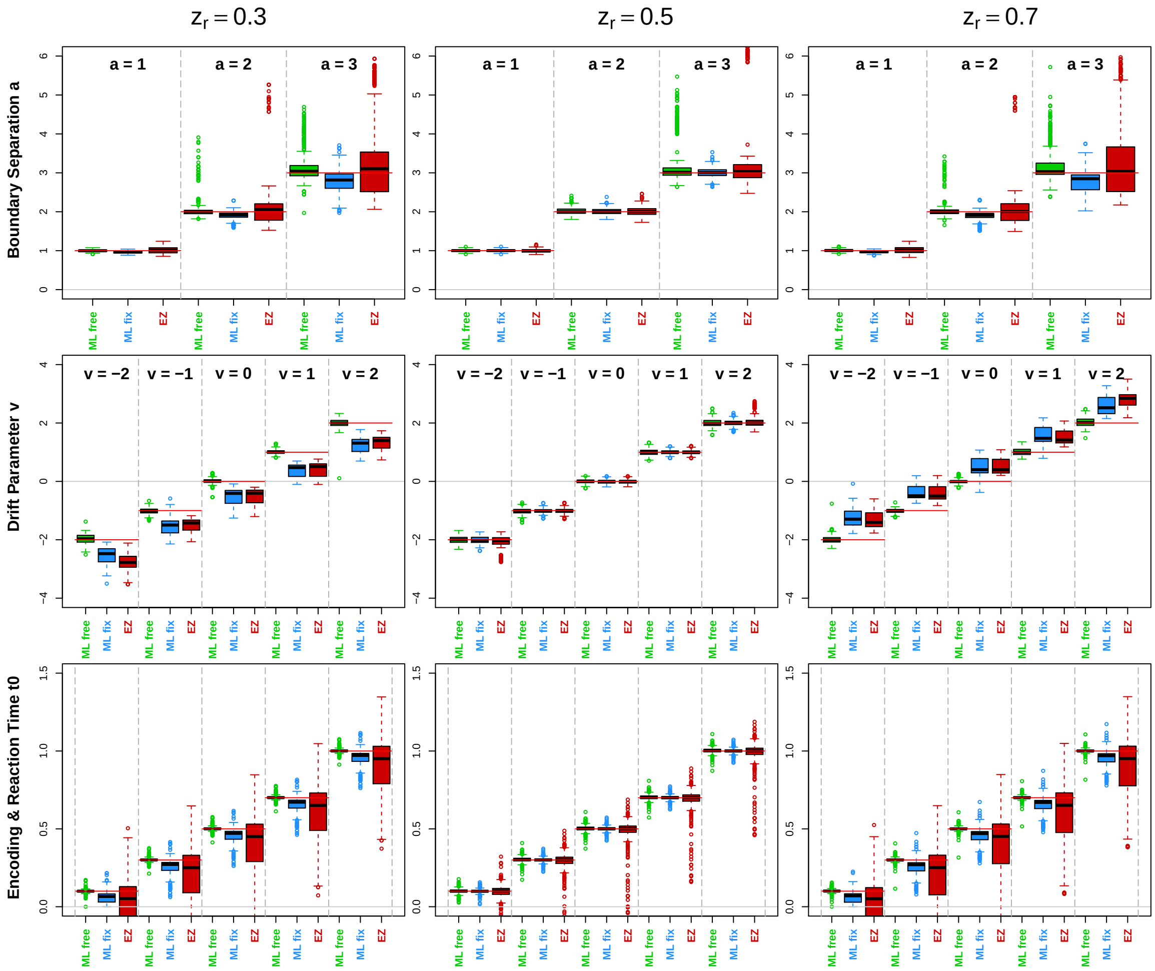

Figure 12: Parameter recovery split by z_{rel}.

First and foremost, we learn from Figure 12 (which is also in the main article) that the ML free method (green) performs excellently in all split groups and for all three parameters. Also, if the true z is 0.5, all three methods produce equal outputs of comparable quality (only the EZ method shows a slightly increased variability of both the $\hat{a}$ and the $\hat{t}_{\text{ER}}$ and the latter yield the negative estimates as observed before).

However, switching to $z \neq 0.5$, we find several peculiarities: The strongest deviations can be found for the $\hat{\nu}$ (middle row), which both ML fix and EZ considerably underestimate for $z = 0.3$ and considerably overestimate for $z = 0.7$. Further, both methods also underestimate the t_{ER}, even dropping below zero with EZ. These effects will be explored parameter-wise in the next Section.

5. Interactions of Parameter Estimates

This section explores, how the estimates of the three parameters a, ν, and t_{ER} change within the various subgroups.

5.1. The Boundary Separation Parameter *a*

5.1.1. ML free

We start with what may be considered the "reference" case, i. e., the unrestricted ML estimation (termed `ML free`). Figure 13 shows the distributions of the estimates $\hat{a}_{\mathrm{ML\ free}}$ split by the true values (a) and the levels of ν and z.

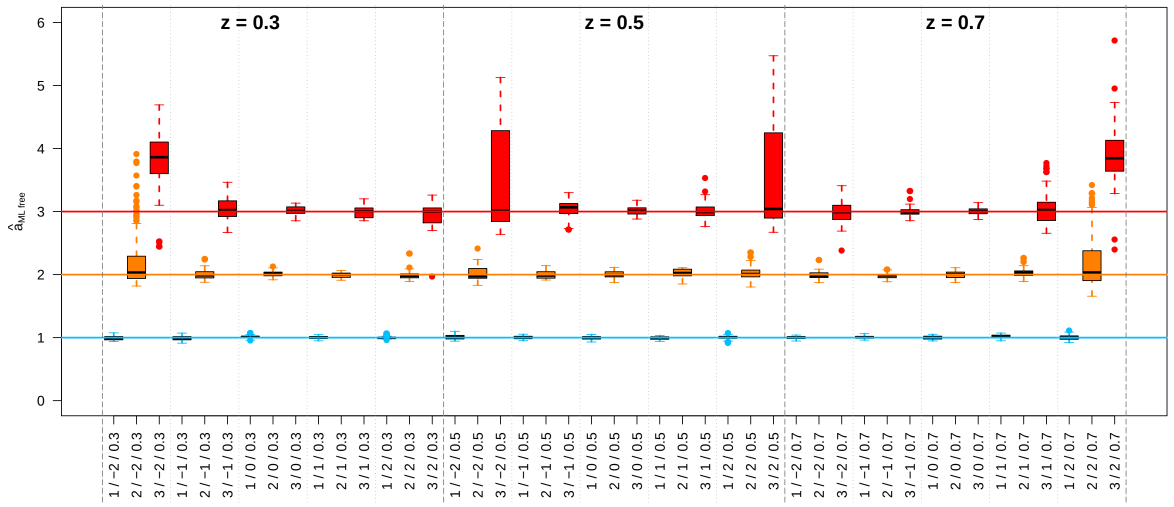

Figure 13: Estimates $\hat{a}_{\mathrm{MLfix}}$ by $a \times \nu \times z$; Notes: Colors indicate the levels of ν (blue: $a = 1$, orange: $a = 2$, red: $a = 3$) and the horizontal lines indicate the true values of a in the same color coding. The dashed vertical lines separate the values of z.

Generally, there seems to be no bias of the $\hat{a}$, but the boxplots indicate some marked outliers and skewed distributions, especially for certain combinations of $a > 1$ and $\nu < 0$. However, these can be explained with the proportion of upper threshold hits (Figure 7).

5.1.2. ML fix

Analysing the same data sets with the ML method but fixing $z = 0.5$ yields the estimates shown in Figure 14:

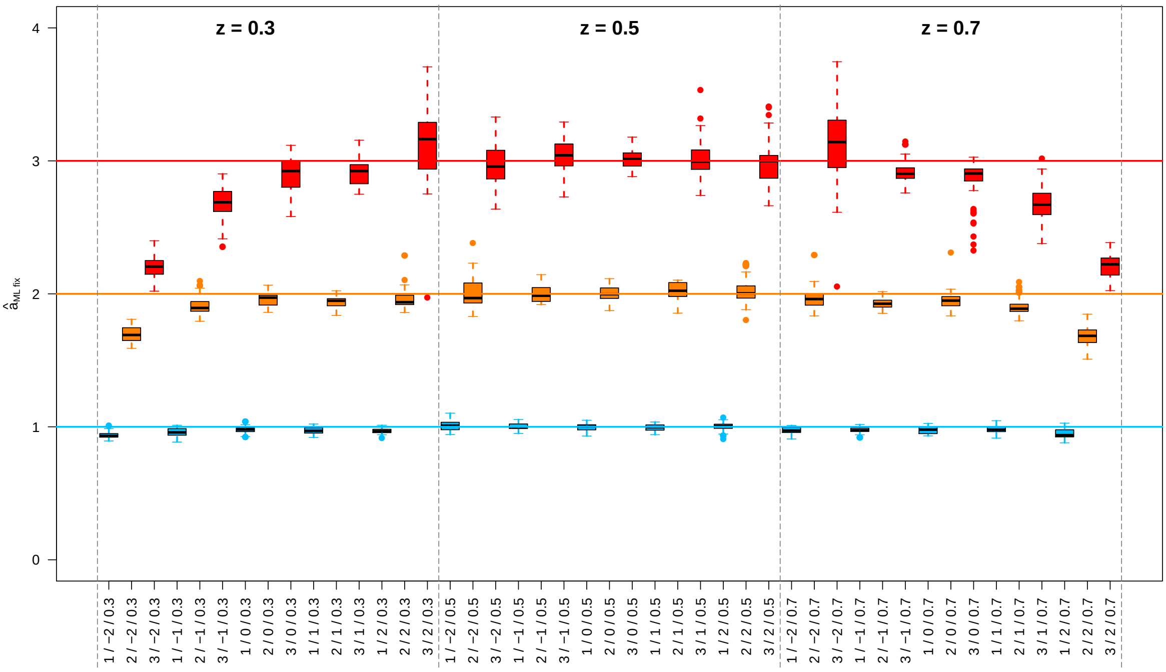

Figure 14: Estimates $\hat{a}_{\text{ML fix}}$ by $a \times \nu \times z$; Notes: Colors indicate the levels of a (blue: $a = 1$, orange: $a = 2$, red: $a = 3$) and the horizontal lines indicate the true values of a in the same color coding. The dashed vertical lines separate the values of z.

This breakdown reveals a far more problematic situation than initially discernible in Figures 6 and (especially) 12. While there is no bias for $z = 0.5$ (middle section) for all three levels of a, the estimates $\hat{a}$ deviate clearly from their true value for $z \neq 0.5$, exhibiting an interaction with ν: The smaller ν for $z = 0.3$ and the larger ν for $z = 0.7$ the larger the downward bias of $\hat{a}$.

Moreover, this effect increases with the value of a: For $a = 1$ (blue), there is almost no bias (just slightly for the most extreme cases $a = 1$, $\nu = -2$ for $z = 0.3$ and $a = 1$, $\nu = +2$ for $z = 0.7$), moderate for $a = 2$ (orange) and remarkably strong for $a = 3$.

5.1.3. EZ

Turning with the same data sets to the EZ method yields the estimates $\hat{a}$ shown in Figure 15:

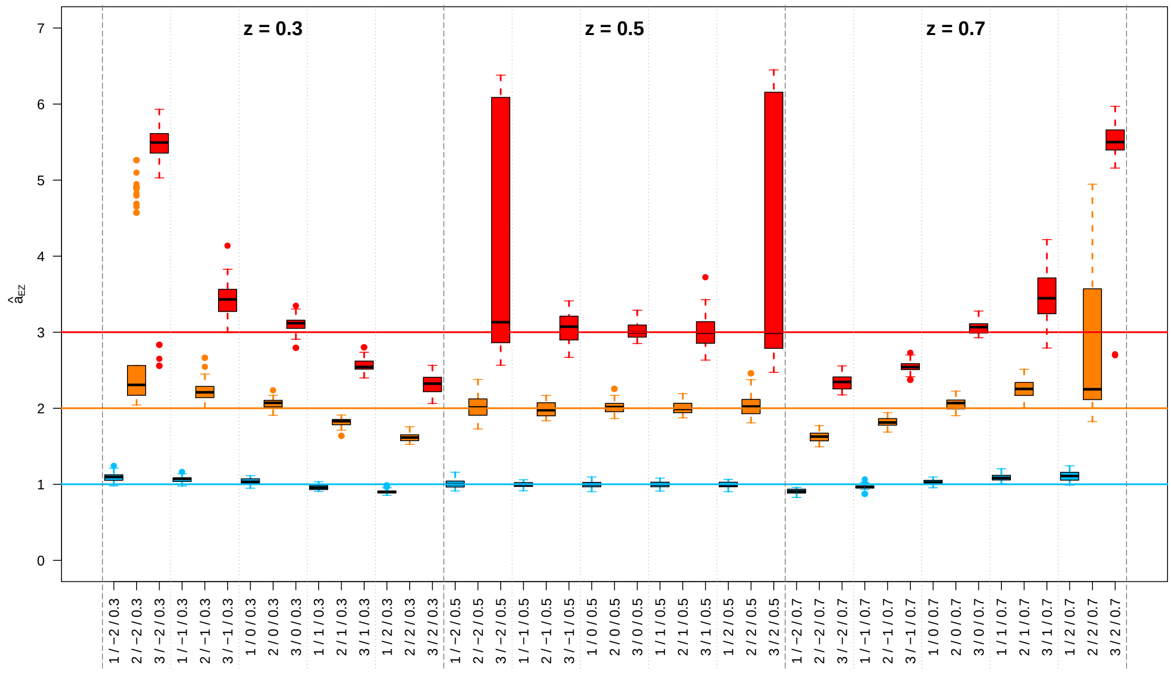

Figure 15: Estimates $\hat{a}_{\text{EZ}}$ by $z \times a \times \nu$; Notes: Colors and horizontal lines indicate the levels of a (see Figure 13).

Here, we find – as before – almost no bias (as indicated by the medians in the boxplots) for the correct case of $z = 0.5$, albeit the heterogeneity of the estimates increases considerably with a and the absolute value of ν.

However, in line with what we have seen for ML fix, also EZ severely misestimates a for $z \neq 0.5$, even stronger and increasing with the value of a. Again, ν and z play a role, but interestingly, and in contrast to ML fix, the effect of ν is reversed, recognizable by the downward tendency of the $\hat{a}$ for $z < 0$ as ν increases and their upward tendency for $z > 0$ as ν increases.

5.2. The Drift Parameter ν

5.2.1. ML free

We start again with the “reference case”. Figure 16 shows the breakdown of the $\hat{\nu}$ by $a \times \nu \times z$.

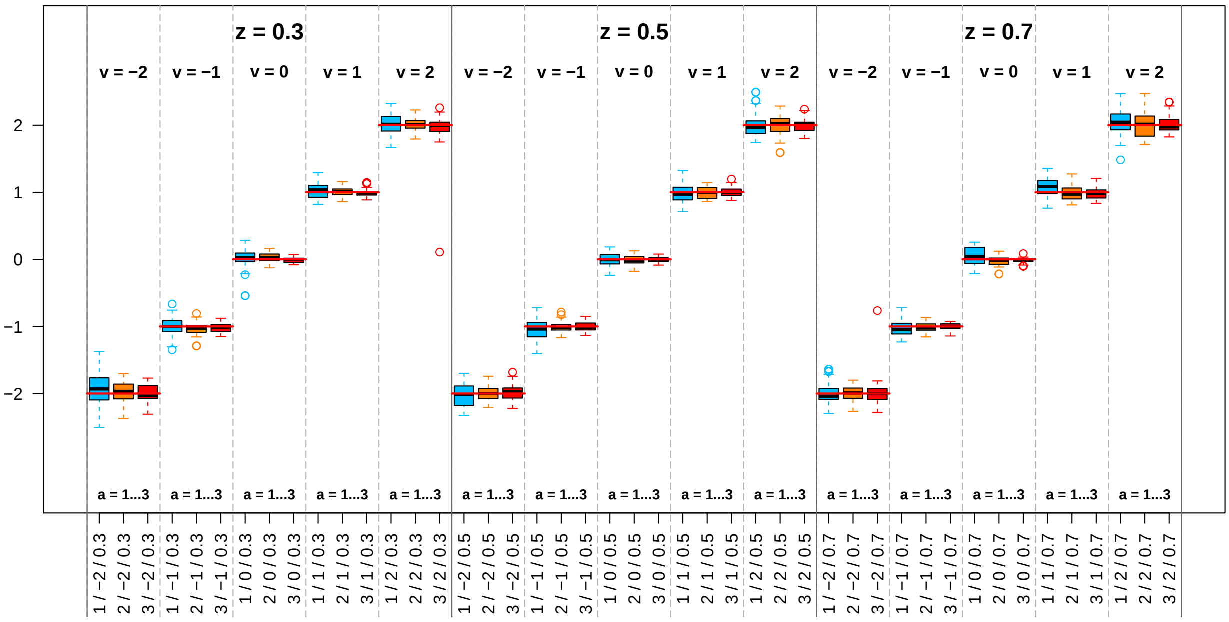

Figure 16: Estimates $\hat{\nu}_{\text{ML free}}$ by $a \times \nu \times z$. Color coding conforms to Figure 13.

For all combinations of a, z, and ν, the ML free estimates of ν were unbiased and had low variability. The ML free method allows for a precise estimation of the drift parameter ν.

5.2.2. ML fix

The marginal distributions of the ML fix estimates $\hat{\nu}$ in Figure 6 was unbiased but exhibited an increased variability compared to the ML free estimates. The breakdown according to a and z reveals the reason for the increased variability (Figure 17).

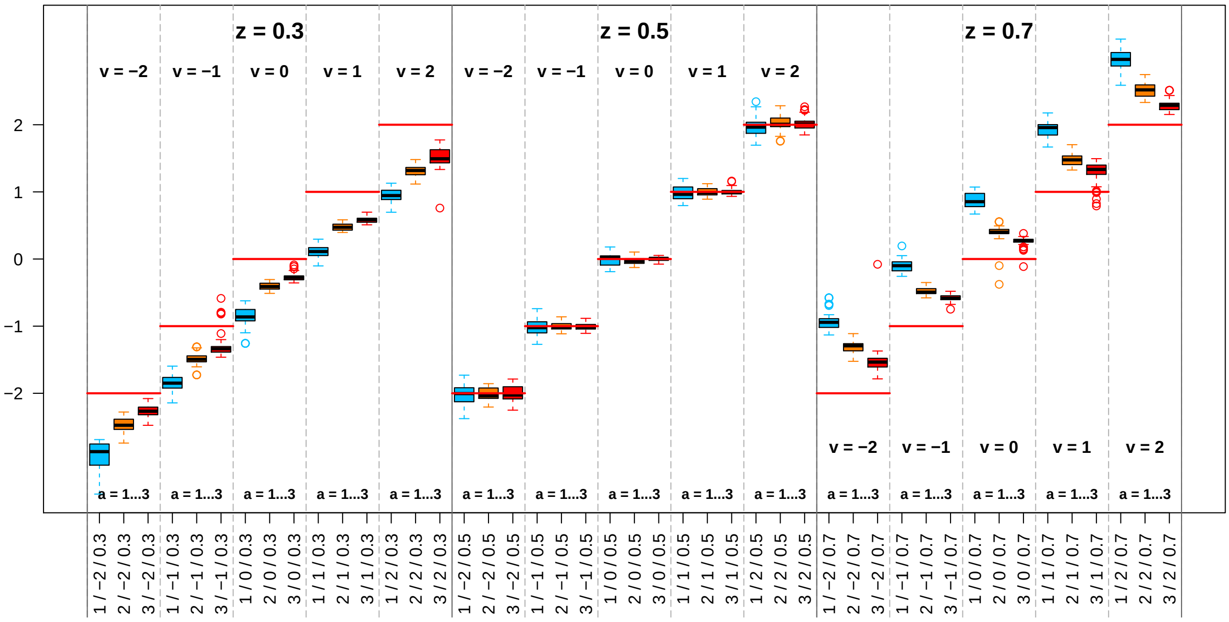

Figure 17: Estimates $\hat{\nu}_{\text{ML fix}}$ by $a \times \nu \times z$. Color coding conforms to Figure 13.

We see that for the correct $z = 0.5$ (middle section), estimates $\hat{\nu}$ are unbiased with low variability, irrespective of the levels of a and ν. However, things change when the true bias is not 0.5: For $z = 0.3$ (left section), we find a systematic (and in several instances severe) underestimation of ν, which decreases as a increases (i. e., it is strongest for $a = 1$ and weakest – yet remarkable – for $a = 3$). In contrast, we find the opposite bias for true $z = 0.7$ (right section), i. e., a positive bias (overestimation), again decreasing with increasing a.

Interestingly, the variability of estimates in the various subgroups is small again. Rather, the opposite directions of bias according to $z < 0.5$ and $z > 0.5$ explain the increased variability of the $\hat{\nu}$ found in the marginal distributions of Figure 6.

5.2.3. EZ

Turning to the EZ estimation method, we find the same phenomena:

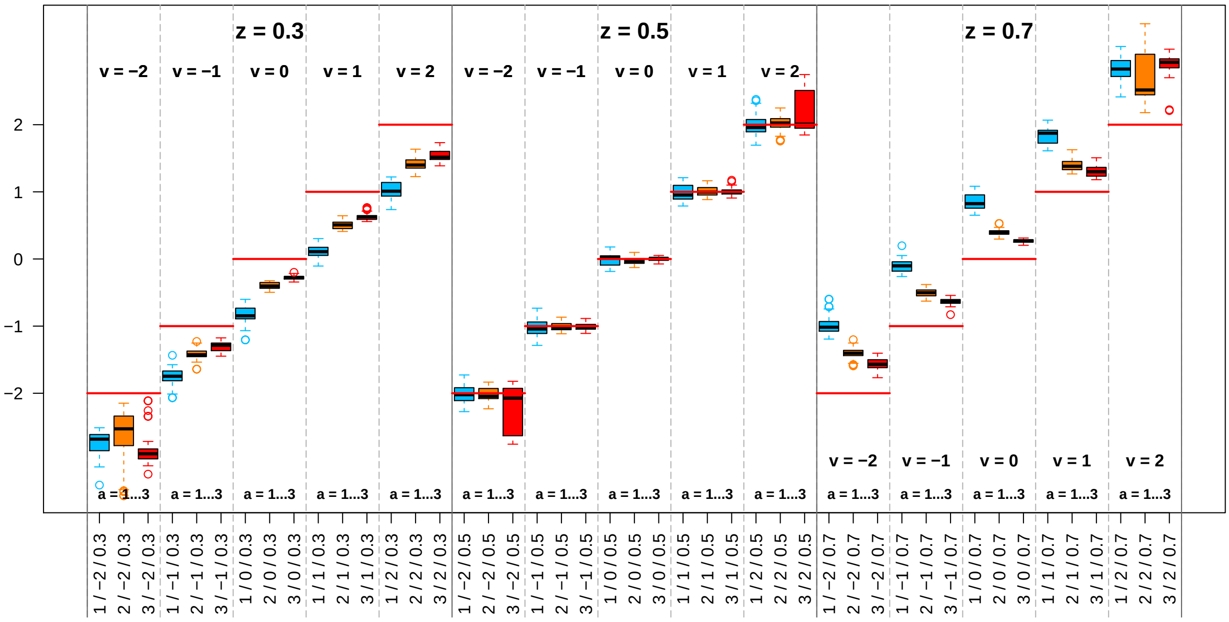

Figure 18: Estimates $\hat{\nu}_{\text{EZ}}$ by $a \times \nu \times z$. Color coding conforms to Figure 13.

Figure 18 shows pretty much the same bias structure as for the ML fix method, except with a somewhat increased heterogeneity in some subgroups. The $\hat{\nu}$ are strictly underestimated for true $z = 0.3$ and overestimated for $z = 0.7$, with a decrease of bias when a increases. Therefore, all conclusions from ML fix apply for EZ as well, it is quite unlikely to obtain correct estimates $\hat{a}$, when z is inadequately fixed at 0.5.

EZ delivers severely biased $\hat{\nu}$ for all $z \neq 0.5$.

5.3. The Encoding and Reaction Time t_{ER}

For analyzing the various estimates of t_{ER}, we switch to the bias $\hat{t}_{\text{ER}} - t_{\text{ER}}$, which allows for keeping the structure of the diagrams as established in the previous sections.

5.3.1. ML free

Again, we start with the reference case ML free (Figure 19):

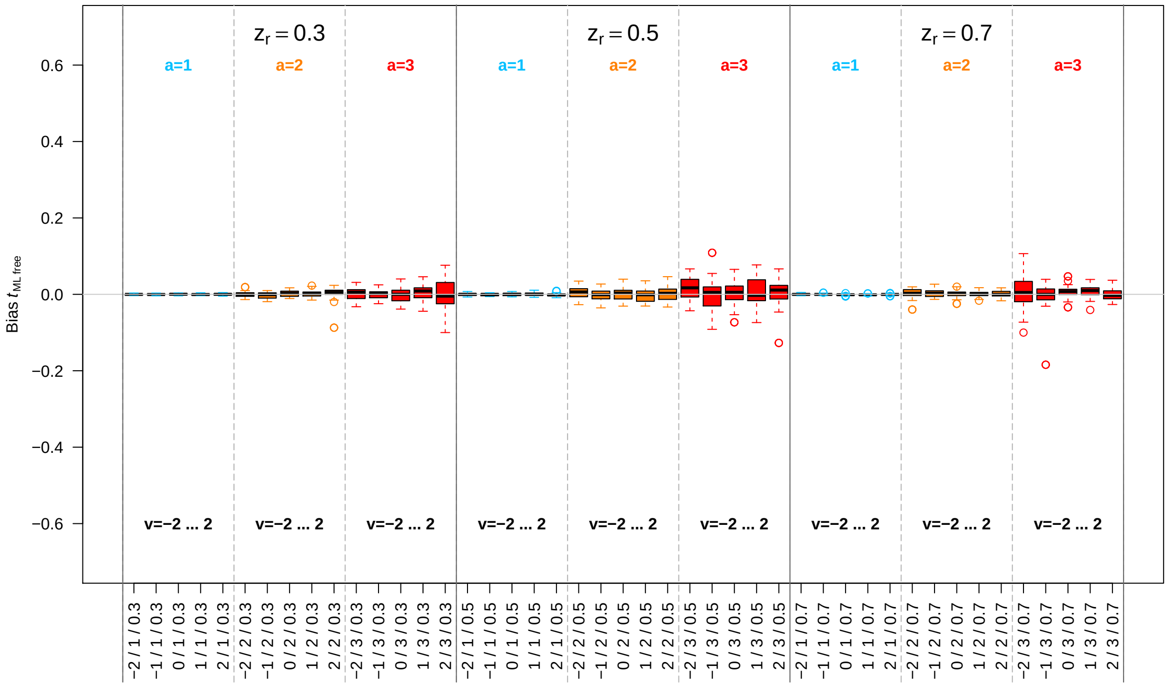

Figure 19: Bias of $\hat{t}_{\text{ML free}}$ by $\nu \times a \times z$. Color coding conforms to Figure 13.

As expected, there is no noticeable bias present in the estimates. Interestingly, the heterogeneity of the $\hat{t}$ (and, hence, the bias) increases somewhat with a. However, these effects appear negligible from a practical point of view.

5.3.2. ML fix

Figure 20 shows the same diagram for the ML algorithm with z fixed at 0.5:

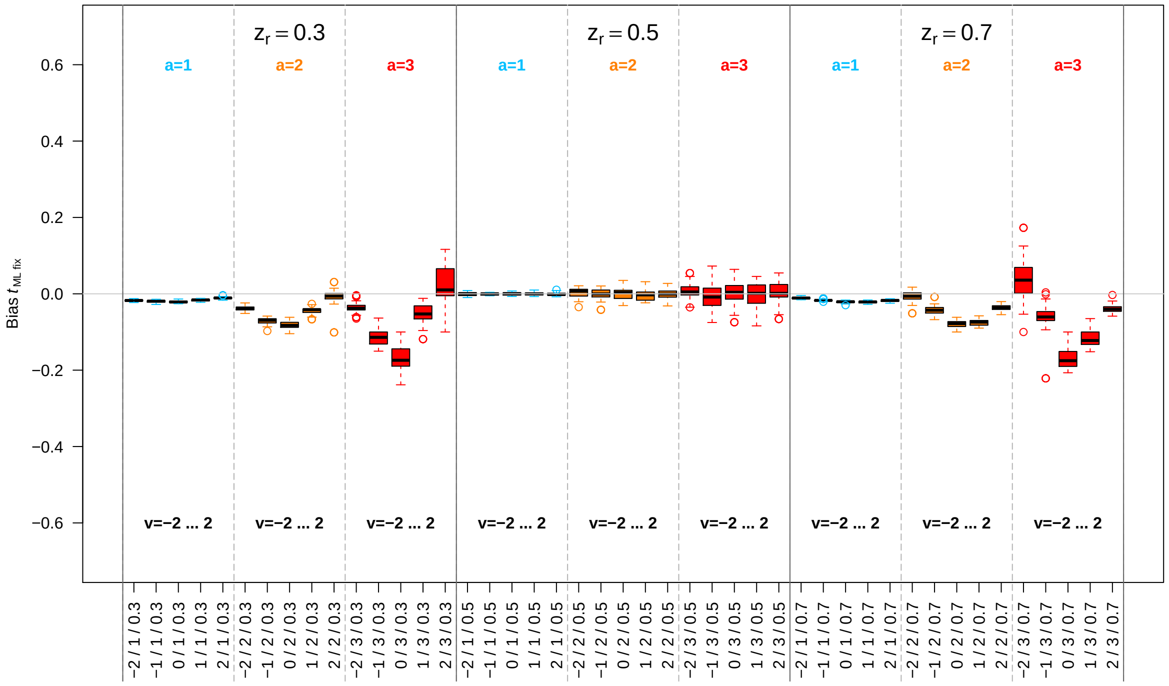

Figure 20: Bias of $\hat{t}_{\text{ML fix}}$ by $\nu \times a \times z$. Color coding conforms to Figure 13.

Here, we find a clear interaction effect of $\hat{t}$ with the drift parameter ν and the boundary separation a if $z \neq 0.5$. Within each segment of Figure 20 formed by the combinations of a and z (dashed vertical lines), we find a U-shaped alignment of the boxplots: The smaller the absolute ν the more negative is the bias of t and this effect increases with a.

5.3.3. EZ

Turning now to the EZ method, we find remarkable deviations (Figure 21):

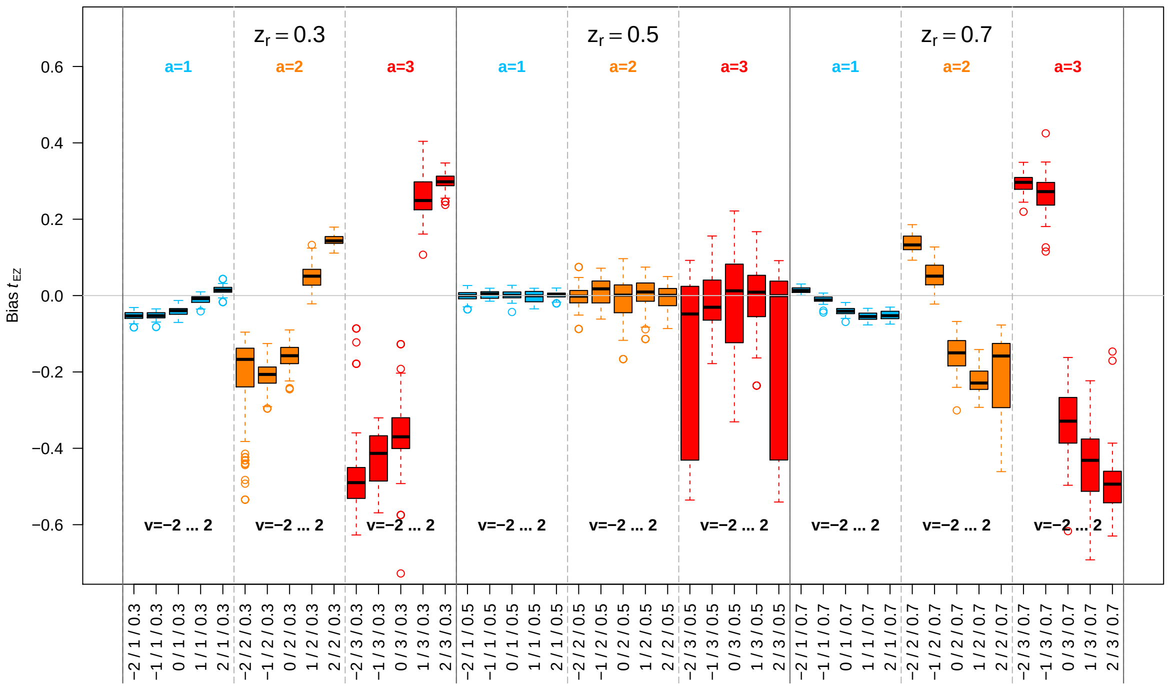

Figure 21: Bias of $\hat{t}_{\text{EZ}}$ by $\nu \times a \times z$. Color coding conforms to Figure 13.

Only for $z = 0.5$, the estimates $\hat{t}$ are more or less unbiased, albeit showing an interaction with ν. For the two most extreme levels of ν, the $\hat{t}$ showed a strong negative bias.

For $z \neq 0.5$, we find severely biased $\hat{t}$: For $z = 0.3$ (left section), the bias of $\hat{t}$ increases visibly with ν and for $z = 0.7$ (right section) it decreases with ν. We might even diagnose a switch from a strong negative to a strong positive biase with the sign of ν (reversed for $z = 0.3$ compared to $z = 0.7$). This effect gets worse the larger a, yielding the inadmissible negative encoding and response times found in the marginal evaluation (Section 4).

For $z \neq 0.5$ the encoding and response time t_{ER} cannot be estimated reliably with the EZ method.

A. The EZ Function

The following script shows the implementation of the EZ routine as provided by Wagenmakers et al. (2007, p. 22), extended by an argument ϵ, which catches the cases $P(+) \in \{0, 0.5, 1\}$:

```
# EZ-algorithm as provided in:
# Wagenmakers, E.-J., van der Maas, H. L. J., & Grasman, R.P.P.P. (2007).
# An EZ-diffusion model for response time and accuracy.
# Psychonomic Bulletin & Review, 14, 3-22.
# -----------------------------------------------------------------------
  ez = function(Pc, MRT, VRT, s=0.1, eps=1e-7) {
       s2 = s^2
       if (Pc %in% c(0,0.5,1)) Pc = Pc + eps - 2*sign(Pc)*eps
       L = qlogis(Pc)
       x = L*(L*Pc^2 - L*Pc + Pc - .5)/VRT
       v = sign(Pc-.5)*s*x^(1/4)
       a = s2*qlogis(Pc)/v
       y = -v*a/s2
       MDT = (a/(2*v)) * (1-exp(y))/(1+exp(y))
       Ter = MRT - MDT
       est = c(a=a,t0=Ter,z=0.5,v=v)
       return(list(par=est))
  } # end of function ez
# -----------------------------------------------------------------------
```

References

Grasman, R. P., Wagenmakers, E.-J., & van der Maas, H. L. (2009). On the mean and variance of response times under the diffusion model with an application to parameter estimation. *Journal of Mathematical Psychology*, *53*(2), 55-68. doi: https://doi.org/10.1016/j.jmp.2009.01.006

R Core Team. (2020). R: A language and environment for statistical computing [Computer software manual]. Vienna, Austria. Retrieved from https://www.R-project.org/

Ratcliff, R. (1978). A Theory of Memory Retrieval. *Psychological Review*, *85*, 59–108.

Singmann, H., Brown, S., Gretton, M., & Heathcote, A. (2020). rtdists: Response time distributions [Computer software manual]. Retrieved from https://CRAN.R-project.org/package=rtdists (R package version 0.11-2)

Wabersich, D., & Vandekerckhove, J. (2014). The RWiener package: An R package providing distribution functions for the wiener diffusion model. *The R Journal*, *6*(1), 49–56. (R package version 1.3-3)

Wagenmakers, E.-J., van der Maas, H. L. J., & Grasman, R. P. P. P. (2007). An EZ-diffusion model for response time and accuracy. *Psychonomic Bulletin & Review*, *14*, 3–22. doi: 10.3758/BF03194023
